# Discontinuous template switching generates coronavirus subgenomic RNAs from the 3ʹ viral genome end by 5ʹ to 3ʹ transcription

**DOI:** 10.1128/jvi.01438-25

**Published:** 2025-09-25

**Authors:** Ayslan Castro Brant, Zhe Hu, Angelika Zelma Chen, Vladimir Majerciak, Jonathan Yewdell, Zhi-Ming Zheng

**Affiliations:** 1 Tumor Virus RNA Biology Section, HIV Dynamics and Replication Program, National Cancer Institute, NIH 585796 , Frederick, Maryland, USA; 2 Cellullar Biology Section, Laboratory of Viral Diseases, National Institute of Allergy and Infectious Diseases, NIH 35037, Bethesda, Maryland, USA; St Jude Children's Research Hospital, Memphis, Tennessee, USA

**Keywords:** coronaviruses, SARS-CoV-2, hCoV-OC43, COVID-19, subgenomic RNA, coronavirus transcription and replication, discontinuous template switch

## Abstract

In coronavirus (CoV)-infected cells, several structural and accessory proteins are synthesized from subgenome RNAs (sgRNA) containing a common genomic 5ʹ-leader followed by a given open reading frame (ORF). We report that the abundance of these sgRNAs varies with distance from the 3′-end of the genome. Thus, there are more sgRNAs encoding nucleocapsid (N) than spike (S), presumably the results from discontinuous 5ʹ−3ʹ transcription template switch mediated by the viral replication and transcription complex (RTC). We optimized the circular polymerase extension reaction (CPER) methodology to generate infectious double-stranded circular cDNA (ds-circDNA) containing the mNeonGreen (NG) reporter in accessory ORFs of human CoVs OC43 and SARS-CoV-2. In each CoV, we found that levels of sgRNAs and NG expression increased with 3ʹ proximal genomic NG location. By reinfection of HCT-8 cells with the same MOI 0.01, however, we found that the slow-growing OC43 NG-ns2 virions exhibited equal infectivity and productivity as the fast-growing OC43 NG-ns12.9 virions. Introduction of point-mutations into the mapped TRS_B_ motif for synthesis of the OC43 ns12.9 and M sgRNAs led to disrupt TRS_B_-TRS_L_ cross-interactions and block production of the corresponding sgRNAs and infectious virions. Together, our data indicate that, by using an optimized CPER approach for positional NG insertions, we demonstrated the progressional reduction of the RTC-mediated template switch (read-through individual TRS_B_) efficiency in production of the corresponding sgRNAs in an order from the 3ʹ viral genome end.

**IMPORTANCE:**

The mechanism for sgRNA synthesis in the coronavirus life cycle is poorly understood. The current model suggests discontinuous template-switch transcription mediated by viral replication and transcription complex (RTC) for synthesis of individual sgRNAs to translate corresponding structural and accessory proteins but lacks experimental data support. This report provides the first experimental evidence that, in both hCoV-OC43 and SARS-CoV-2, viral RTC synthesizes its sgRNAs by long-range base-pairing between a distal transcription regulatory body sequence (TRS_B_) upstream of each structural/accessory ORF and the transcription regulatory leader sequence (TRS_L_) from the viral genome 5ʹ-UTR, leading to the production of viral sgRNAs in abundance order from the viral genome 3ʹ -end, with more N sgRNAs but less S sgRNAs. Our data support a “first-come, first-serving” model in TRS_B_-TRS_L_ cross-interaction and read-through TRS_B_ process to mediate discontinuous transcription switch in coronavirus sgRNA synthesis in a 5ʹ−3ʹ transcription direction from the 3ʹ viral genome during coronavirus infection.

## INTRODUCTION

Coronaviruses infect many mammalian species, including humans. Four of the seven known human coronaviruses (hCoV) (hCoV-229E, -HKU1, -NL63, and -OC43) generally cause mild upper respiratory infections, while three (SARS-CoV, MERS-CoV, and SARS-CoV-2) can cause severe acute respiratory syndrome (SARS) (1–3). Coronaviruses are divided into Alpha-, Beta-, Gamma-, and Delta-genera. hCoV-229E and hCoV-NL63 belong to the Alpha-coronavirus, and hCoV-OC43, hCoV-HKU1, SARS-CoV, MERS-CoV, and SARS-CoV-2 belong to the Beta-coronavirus. Despite hCoV-NL63 and SARS-CoV-2 belonging to different genera, they utilize the same cellular receptor, the angiotensin 1-converting enzyme 2 (ACE2) to enter cells (2).

The coronavirus genomic RNA (gRNA) is a single-stranded positive-sense 26–32 kb RNA. The gRNA, which encodes 20 to 29 known viral proteins (4–6), has a 5ʹ-end cap followed by a 5ʹ untranslated region (5ʹ-UTR), a long coding region, and a 30–60 nt 3ʹ-end poly-A tail. Approximately 70% of the viral genome encodes ORF1a and ORF1b, which are translated into two polyproteins post-translationally cleaved into the 16 viral nonstructural proteins (nsps) (2). The remaining 30% of the genome encodes structural (S, E, M, and N) and accessory proteins. The number of accessory proteins varies among coronaviruses, and their functions are poorly defined but presumably contribute to viral pathogenesis (7–9) as most are nonessential for viral replication in cultured cells (7, 10–12). The viral genome 5ʹ- UTR contains a 72-nt leader, a transcription regulatory sequence motif (TRS_L,_ ACGAAC), and other translation and genome package regulatory cis-elements. A transcription regulatory body sequence (TRS_B_) upstream of each structural or accessory ORF region is presumed to control subgenome RNA (sgRNA) synthesis (13, 14), which is robust. The sgRNAs are excluded from virions via unknown mechanisms.

During viral penetration, the +gRNA is released into the cytosol where ORF1a and ORF1b are translated into a polyprotein cleaved into 16 nsps. The nsp7, nsp8 (×2), nsp9, nsp12, and nsp13 (×2) associate with a viral +gRNA template and an RNA primer to form the Replication and Transcription Complex (RTC) (15, 16). Inside the virus-induced double-membrane vesicles (DMVs), the RTC synthesizes negative-strand genomic RNA (-gRNA) and a subset of negative-strand sgRNAs (-sgRNAs) from a viral +gRNA template (17, 18). The -gRNA and -sgRNAs are then used as a template separately to synthesize full-length +gRNA for virion generation and +sgRNAs for translation of a given structural or accessory protein (2).

The mechanism for -sgRNA synthesis is poorly understood. A current model suggests discontinuous transcription mediated by RTC template switching by long-range base-pairing between distal TRS_B_ of the 6–7 nt core sequence and the TRS_L_ motif from the viral genome 5ʹ-UTR (2, 5, 14, 19–21). As the RTC synthesizes the -sgRNAs from the +gRNA 3ʹ-end in the 5ʹ−3ʹ direction, it may temporarily dissociate the template +gRNA at TRS_B_ to enable the RTC to translocate to the TRS_L_ leader, skipping a large fraction of the viral genome in this template switch. By going through individual TRS_B_ for the template switch, this generates variably sized -sgRNAs, of which further serve as individual templates synthesizing the corresponding +sgRNAs whose first ORF is translatable (2, 21).

The aim of this study is to better understand the proposed discontinuous transcription template switch model (2) by comparing the sgRNA synthesis of two human coronaviruses hCoV-OC43 and SARS-CoV-2. We employed an autofluorescent mNeonGreen (NG) reporter protein (22) to generate a set of recombinant infectious viruses using an optimized circular polymerase extension reaction (CPER) (23–25). By positional NG insertion to an accessory ORF (7, 10–12), we provide compelling evidence supporting a “first-come, first-serving” model in TRS_B_-TRS_L_ cross-interaction-mediated discontinuous transcription for coronavirus sgRNA synthesis (2).

## RESULTS

### Optimizing CPER to construct SARS-CoV-2 and hCoV-OC43 double-stranded circular cDNAs (ds-circDNA)

We optimized the CPER-based system originally devised to generate flavivirus (23) and subsequently, SARS-CoV-2 (24, 25) infectious clones. We obtained the linker plasmid from Alberto A. Amarilla and Alexander A. Khromykh (25), which is a central component for efficient CPER. By PCR introduction of a 20 bp nucleotide sequence on the linker 5ʹ-end and a 37 bp sequence on the linker 3ʹ-end, respectively, to overlap the corresponding viral genome 3ʹ-UTR end and 5ʹ-UTR end (Fig. S1A), we made the linker specific for construction of individual infectious ds-circDNAs of hCoV-OC43 and SARS-CoV-2.

**Fig 1:**
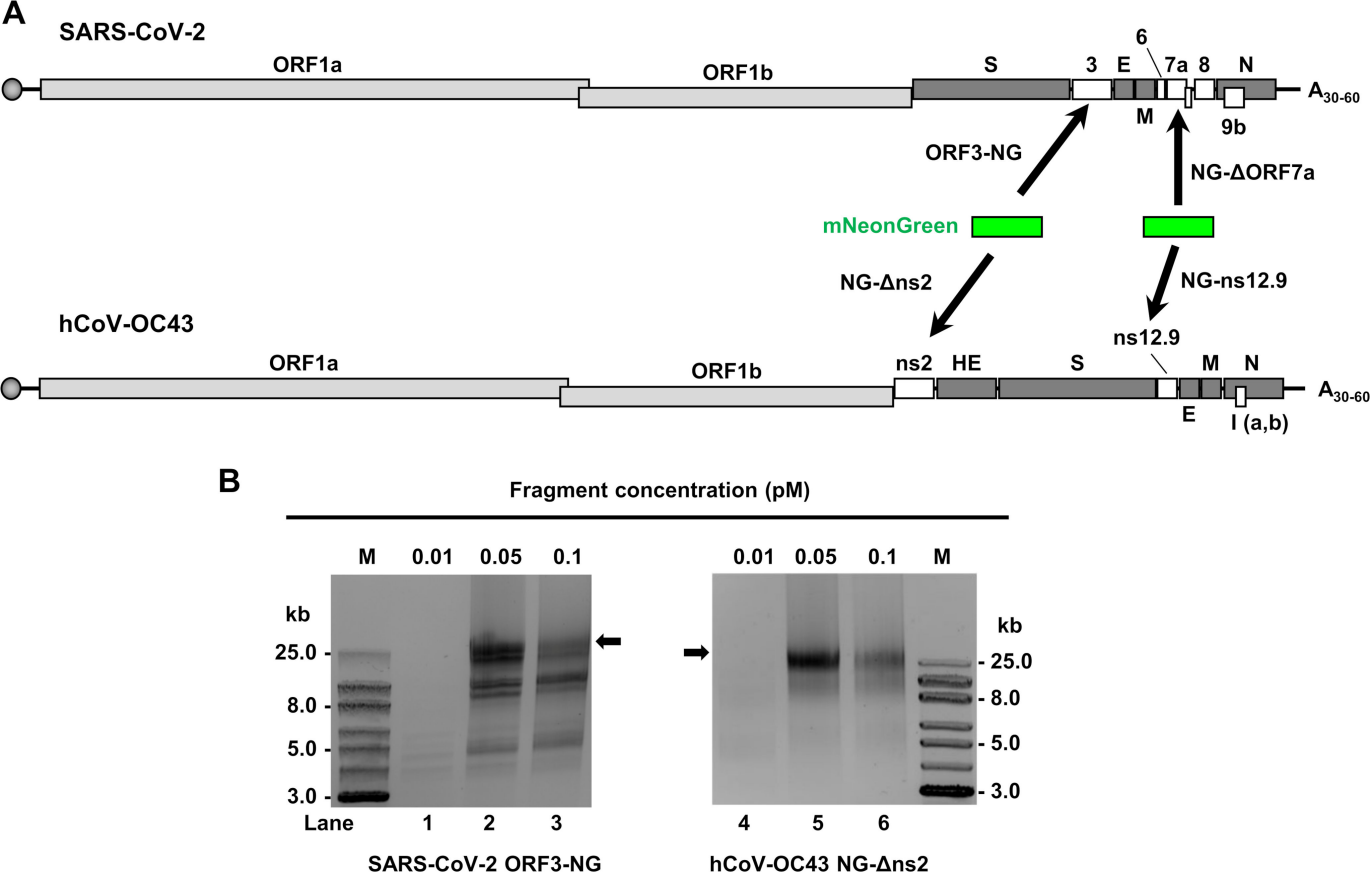
Insertion of an mNeonGreen (NG) report into a coronaviral accessory protein ORF to produce CPER-derived full-length (FL), double-stranded circular cDNA (ds-circDNA). (**A**) Diagrams show where the NG was positionally inserted into an accessory protein ORF (white box) of individual coronavirus genomes. (**B**) Agarose gel (0.8%) electrophoreses to determine the optimal concentration of each cDNA fragment and the linker for production of CPER-derived ds-circDNAs. Three different concentrations of each cDNA fragment were tested (0.01 pM, 0.05 pM, and 0.1 pM) during CPER, and the determined optimal concentration for production of a viral FL genome product (black arrow) was 0.05 pM for SARS-CoV-2 ORF3-NG (lane 2) and hCoV-OC43 NG-Δns2 (lane 5). M, DNA kb markers

Seven overlapping DNA fragments (F1 to F7) were amplified by PCR from the full-length (FL) SARS-CoV-2 (26) and hCoV-OC43 cDNAs (Fig. S1B, steps 1 and 2, Fig. S2 and Table S1), with 20–40 nt overlapping from each fragment. We took viral accessory ORF in a given cDNA fragment for insertion of an autofluorescent NG reporter by overlapping PCR (Fig. 1A; Fig. S2). By annealing overlapped cDNA fragments with the virus-specific linker (Fig. S1B, step 3), we generated semi-circular cDNA through base-pairing from the overlapped sequences, with each fragment acting as a Taq DNA polymerase template/primer to fill inter-fragment gaps (Fig. S1B, step 4). The final product is a virus-specific ds-circDNA (Fig. S1B, step 5).

We determined the optimal fragment concentration for individual CPER reactions by using 0.01, 0.05, and 0.1 pM of individual fragments to generate FL ds-circDNAs with a NG insertion, as determined by agarose gel electrophoresis (Fig. 1B). This revealed that the CPER reaction with 0.05 pM of each cDNA fragment displayed the best production of >25 kb ds-circDNA band (Fig. 1B, lanes 2 and 5, black arrows), corresponding to a FL cDNA of the SARS-CoV-2 or hCoV-OC43 genome.

### Optimizing CPER-derived hCoV-OC43 NG-virus production

**Fig 2:**
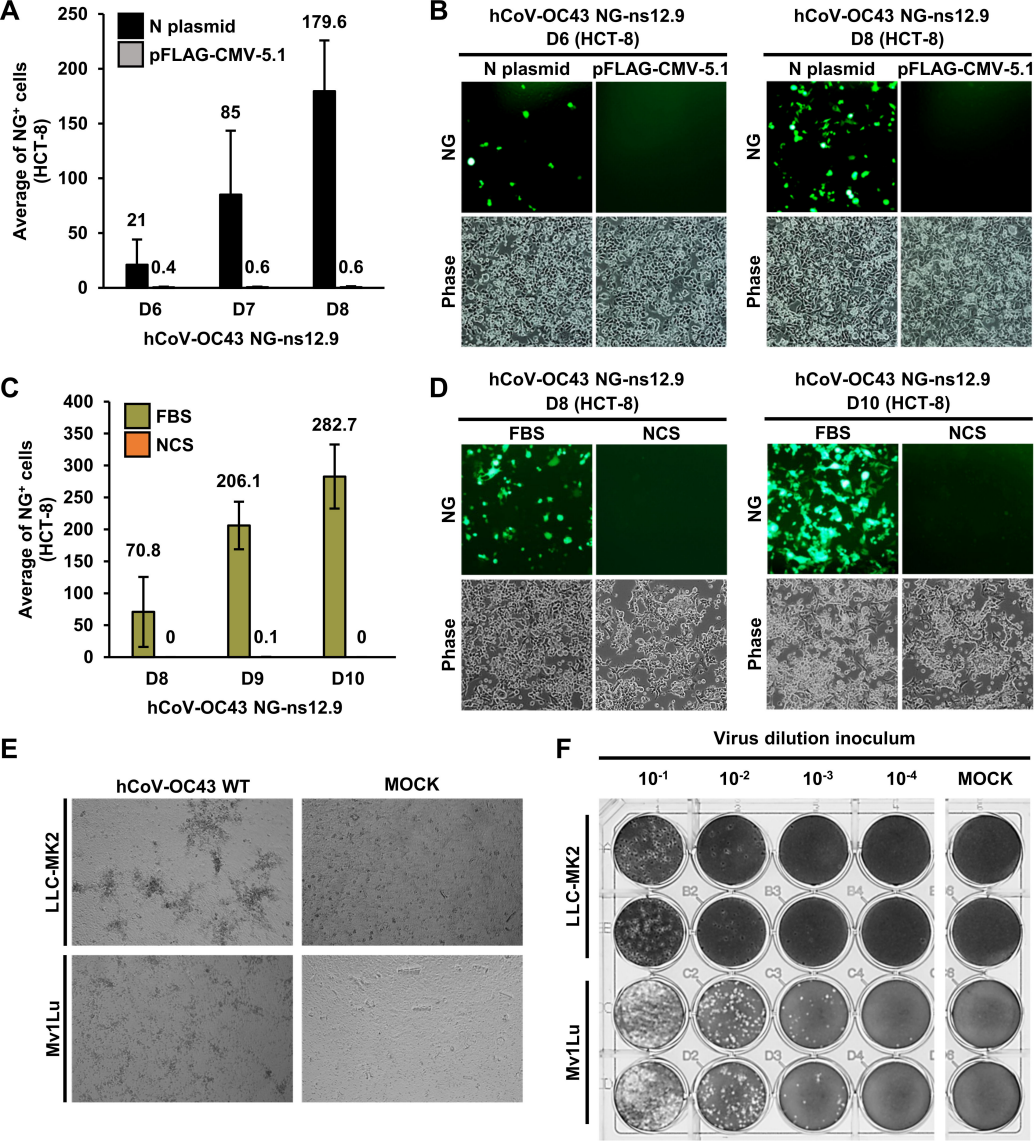
Optimizing hCoV-OC43 NG-ns12.9 ds-circDNA transfection and co-cultivation for infectious NG virus production. (**A**) The N protein promotes virus production from CPER-derived ds-circDNAs. HEK293T cells were co-transfected with an FL hCoV-OC43 NG-ns12.9 ds-circDNA along with an hCoV-OC43 N expression vector (pCOC42) or a control vector (pFLAG-CMV-5.1) and then co-cultivated with HCT-8 for 8 days. The numbers of NG^+^ HCT-8 cells were counted and averaged from 10 random microscopic fields on days 6, 7, and 8 (D6–D8) post-transfection. (**B**) A representative microscopic field showing the NG^+^ HCT-8 cells co-transfected by an FL hCoV-OC43 NG-ns12.9 ds-circDNA with an hCoV-OC43 N expression vector (pCOC42) or a control vector pFLAG-CMV-5.1 on D6 and D8. (**C, D**) Fetal bovine serum (FBS), but not new calf serum (NCS), is required for efficient virus production in HCT-8 cells. HEK293T cells were co-transfected with an FL hCoV-OC43 NG-ns12.9 ds-circDNA along with an N expression vector pCOC42 and maintained in DMEM supplemented with 2% FBS overnight. The transfected cells were then co-cultivated by the addition of HCT-8 cells with cell passage every 3 days for a total of 10 days in DMEM supplemented with 10% FBS or 10% NCS. The number of NG^+^ HCT-8 cells was counted and averaged from 10 random microscopic fields on D8–D10 (**C**). Significantly more NG^+^ HCT-8 cells were shown from the cells with an FL NG-ns12.9 ds-circDNA on D8–D10 when growing in the DMEM containing 10% FBS when compared with 10% NCS (**D**). (**E**) hCoV-OC43 induced visible and well-defined cytopathic effect (CPE) in both LLC-MK2 and Mv1Lu cells. The monolayer of LLC-MK2 or Mv1Lu cells in ~70% confluence was infected with WT hCoV-OC43 (100 µL supernatant of infected HCT-8 cells). One representative microscopic field is shown for each cell type. (**F**) Mv1Lu cells are more sensitive than LLC-MK2 cells for hCoV-OC43 infection and plaque formation. Plaque assays of LLC-MK2 and Mv1Lu cells were infected with 100 µL of each diluent after serial 10-fold dilutions of WT hCoV-OC43 virus and overlayed with semisolid media (1× DMEM, 0.5% methylcellulose and 10% FBS) for 8 days. The plaques were fixed for 30 min by 3.7% formaldehyde solution and stained with 1% crystal violet.

We first examined CPER-derived FL ds-circDNAs of hCoV-OC43 NG-ns12.9 by co-transfecting HEK293T cells with either a hCoV-OC43 N protein expression plasmid (pCOC42) or empty control plasmid pFLAG-CMV-5.1, and incubating the transfected cells for 24 h (Fig. S1C) before adding HCT-8 cells. We observed a constant increase of NG^+^ cells when we co-transfected cells with the N plasmid pCOC42, but not with the pFLAG-CMV-5.1 (Fig. 2A and B). Thus, CPER-derived ds-circDNA co-transfection with the N protein expression is essential for efficient virus production.

We next examined the serum requirement for infectious virus generation. We compared fetal bovine serum (2% FBS) to newborn calf serum (2% NCS, a cheaper alternative to FBS) for transfected cell cultures in production of infectious viruses at 33°C from the co-transfected HEK293T and co-cultivated with HCT-8 cells. We found there was a progressive increase in the number of NG^+^ cells in 2% FBS cultivated cells, starting from ~70 NG^+^ cells/field on day 8 (D8) to ~282 NG^+^ cells/field on day 10 (D10), but not the cells under a 2% NCS culture condition (Fig. 2C and D). The data indicate that FBS was essential for successful propagation of the recombinant infectious virus that originates from the ds-circDNAs of hCoV-OC43 NG-ns12.9 constructed by the CPER technology.

Despite the efficient replication, hCoV-OC43 infection of HCT-8 cells does not lead to visible plaques, a traditional method to determine the virus titer. To identify a cell line suitable for hCoV-OC43 plaque assay and titration of our CPER-derived infectious clones, we confirmed both LLC-MK2 (monkey kidney cells) and Mv1Lu (Aleutian mink lung cells) are susceptible to wild-type (WT) hCoV-OC43 infection (Fig. 2E) (27–30). By comparing their susceptibility for plaque formation using WT hCoV-OC43 produced by HCT-8 cells, we found that Mv1Lu cells are more permissive for hCoV-OC43 plaque formation than LLC-MK2 cells, with a 100-fold higher titer by this assay (Fig. 2F). We therefore used Mv1Lu cells to titer CPER-derived infectious NG-hCoV-OC43.

### CPER-derived hCoV-OC43 NG-ns12.9 ds-circDNA generates more NG^+^ viruses than CPER-derived hCoV-OC43 NG-Δns2 ds-circDNA

**Fig 3:**
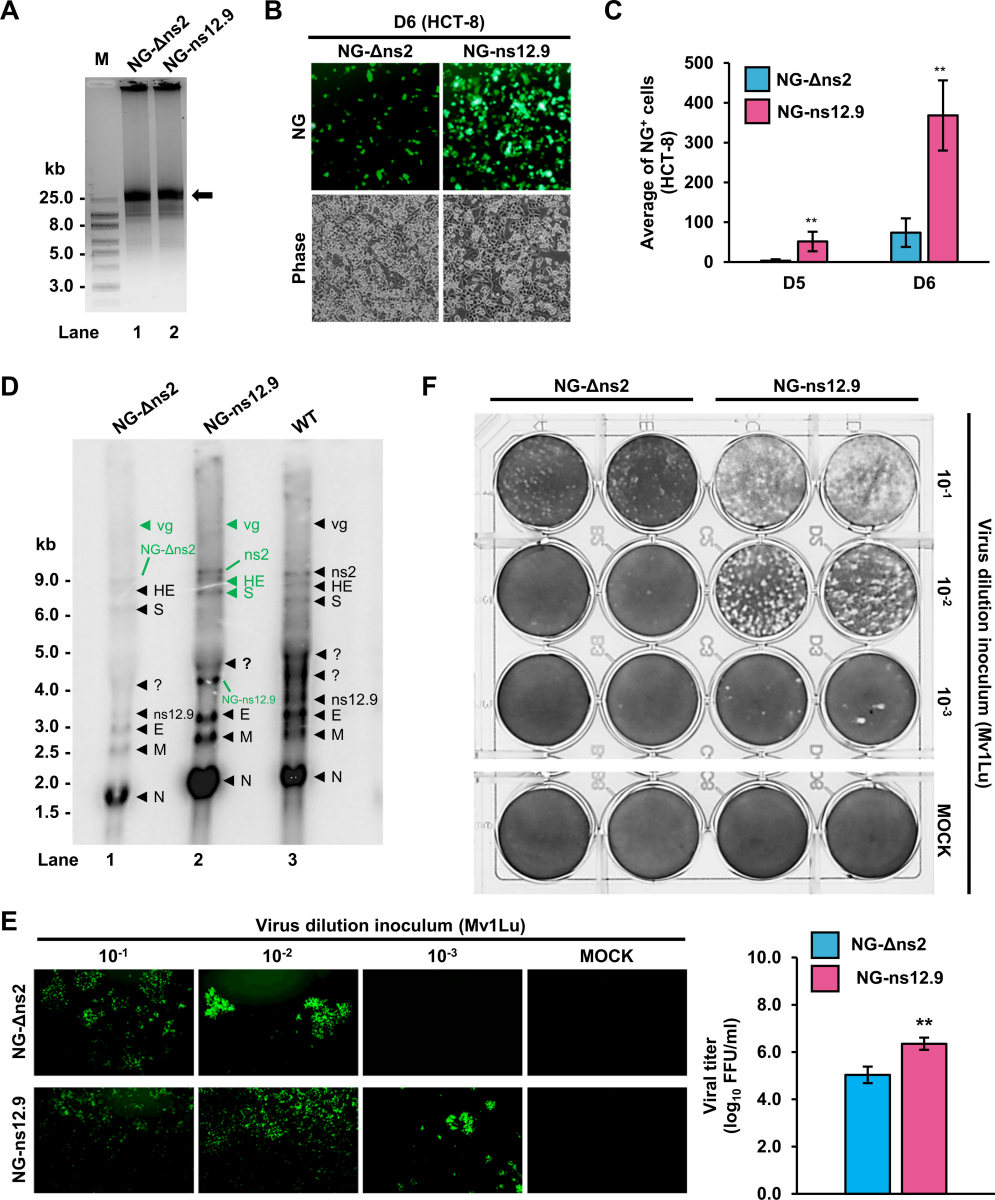
Positional NG insertion into the hCoV-OC43 genome to determine hCoV-OC43 replication and synthesis direction of viral sgRNAs. (**A**) Agarose gel electrophoresis of CPER-derived hCoV-OC43 ds-circDNAs (black arrow) of NG-Δns2 (lane 1) and NG-ns12.9 (lane 2). See Fig. 1A for details. (**B and C**) Visualization and quantification of NG^+^ HCT-8 cells after co-cultivation with HEK293T cells transfected with CPER-derived hCoV-OC43 NG-Δns2 or NG-ns12.9 ds-circDNAs. HEK293T cells were transfected with individual FL ds-circDNA products along with an hCoV-OC43 N protein expression vector pCOC42 and then co-cultivated by addition of HCT-8 cells for the indicated days. (**B**) Representative microscopic images on D6 showing NG^+^ HCT-8 cells from the NG-Δns2 or NG-ns12.9 ds-circDNA. (**C**) Numbers of NG^+^ cells quantified from 10 random microscopic fields on D5 and D6, showing more NG^+^ cells (**, *P* < 0.01, Student’s *t*-test) for the NG-ns12.9 ds-circDNA than that of the NG-Δns2 ds-circDNA. (**D**) Northern blot analysis of hCoV-OC43 RNA from HCT-8 cells in co-cultivation with HEK293T cells transfected by CPER-derived NG-Δns2 ds-circDNA (lane 1) or NG-ns12.9 ds-circDNA (lane 2) or infected by wild-type (lane 3) viruses. Total RNA extracted from the D6 cells was analyzed by Northern blot using a ^32^P-labeled probe antisense to the hCoV-OC43 N ORF. The sgRNAs containing inserted NG are labeled in green. (**E, F**) Virus titration of the HCT-8 cell culture supernatant collected on D8 co-cultivation by using methylcellulose-overlay fluorescent foci (**E**) and plaque (**F**) assays in Mv1Lu cells. (**E**) Representative images of fluorescent foci on D8 of infected Mv1Lu cells (left panel). The Mv1Lu cells were infected by 100 µL of each diluent after serial 10-fold dilutions of the collected HCT-8 culture supernatant collected on D6 described above (**C**). Virus titers are calculated as fluorescent-forming units (FFU/mL) (right bar graph), showing a higher titer of hCoV-OC43 NG-ns12.9 virus (1.2 × 10^6^ FFU/mL) than hCoV-OC43 NG-Δns2 virus (4.0 × 10^4^ FFU/mL). (**F**) Titration of hCoV-OC43 NG-Δns2 and NG-ns12.9 virus titers by plaque assays using Mv1Lu cells infected by 100 µL of each diluent after serial 10-fold dilutions of the HCT-8 culture supernatant collected on D6 of the cell co-cultivation. The visible virus plaques of hCoV-OC43 NG-Δns2 and hCoV-OC43 NG-ns12.9 on D8 in Mv1Lu cells were visualized by crystal violet staining.

After establishment of the optimal conditions for CPER-based system, we next examined if a positional insertion of the NG reporter into hCoV-OC43 would affect virus replication efficiency. hCoV-OC43 contains two accessory ORFs, an ns2 (31) toward the 5ʹ half of the virus genome and an ns12.9 (32) toward the 3ʹ half of the viral genome (Fig. 1A). As expected, deletion or NG insertion of the ns2 was not detrimental to hCoV-OC43 virus infection and replication (33) (Fig. S2B and S3). Parallel co-transfection of CPER generated FL ds-circDNA products (Fig. 3A, black arrow) of hCoV-OC43 NG-Δns2 or NG-ns12.9 with the N plasmid pCOC42 into HEK293T cells for 24 h was followed by addition of HCT-8 cells for co-cultivation for the indicated days. To our surprise, we observed that the cells with transfected NG-ns12.9 ds-circDNA showed a consistently higher number of NG^+^ cells than NG-Δns2 ds-circDNA transfection (Fig. 3B and C). The NG-ns12.9-transfected cells produced almost five times more NG^+^ cells than NG-Δns2 both at D5 and D6 (Fig. 3C). These data were highly reproducible, confirming a higher transcription and replication capability of the NG-ns12.9 infectious clone with an NG insertion toward the 3ʹ-half of the virus genome than the NG-Δns2 infectious clone with an NG insertion toward the 5ʹ-half of the virus genome.

To validate this observation, we isolated total cell RNA from the co-cultured cells on D6 for detection of viral RNA transcripts by Northern blot and collected the culture supernatants for virus titration by the plaque assay described in Fig. 2F. Using a ^32^P-labeled anti-sense oligo probe from the hCoV-OC43 N ORF region capable of detecting viral +gRNA and all +sgRNAs, we examined the equal amount of the extracted total cell RNA from each transfection and demonstrated, as expected, the higher amount of viral RNA transcripts from the NG-ns12.9-transfected cells, comparable to WT hCoV-OC43-infected cells, than that from the NG-Δns2-transfected cells (Fig. 3D, compare lanes 2 and 3 to 1). The data further indicated the preferable higher viral genome transcription and replication of the NG-ns12.9 than the NG-Δns2. We also observed the expected RNA size increase from the inserted NG reporter by 762 nt for the individual sgRNAs S, HE, ns2, and the viral gRNA of NG-ns12.9 (green labels) (Fig. 3D, compare lines 2 to 3, and Table S2). In the NG-Δns2, the size shift of NG-Δns2 sgRNAs and viral gRNA (green labels) was only minimal with only an additional 6 nt difference from the corresponding WT virus sgRNAs and gRNA, as NG insertion in the NG-Δns2 genome compensated the partial deletion of the ns2 (Fig. 3D, compare lanes 1 and 3, and Table S2).

The increased virus replication resulting in higher virus titer of the CPER-derived NG-ns12.9 than that from the NG-Δns2 was further verified by plaque assays using Mv1Lu cells (Fig. 3F). Mv1Lu cells were infected with cell-free virions released to the culture supernatants on D6 and overlaid with semisolid DMEM-methylcellulose overlay media for up to 8 days. By calculation of fluorescent-forming units (FFU) from the infected Mv1Lu cells under fluorescent microscopy, we observed higher FFU appearance in the NG-ns12.9-infected cells than that in the NG-Δns2-infected cells, with FFU on D8 reaching 1.2 × 10^6^ FFU/mL for the NG-ns12.9, while only 4.0 × 10^4^ FFU/mL for the NG-Δns2 (see Fig. 3E, the left for FFU images and the right for bar graph). Crystal violet staining of a fixed monolayer in the titration plate confirmed a higher replication titer of the NG-ns12.9 than the NG-Δns2 (Fig. 3F) in the plaque assays.

Total cell RNA-seq and viral gRNA sequencing indicated that both OC43 NG viruses, NG-ns12.9 and NG-Δns2, exhibited the same three mutations from the ATCC WT hCoV-OC43 (GenBank accession no. AY391777), of which are reverted to the sequence observed in another reference genome (GenBank accession no. NC_006213.1). These mutations are at viral genome positions nt 32U-to-C in the virus leader and nt 26997G-to-C and nt 27018C-to-U in the S ORF (Table S4). The nt 26997G-to-C causes a change of methionine to isoleucine. The nt 27018C-to-U is silent mutation. Data indicate that the observed difference in viral transcription and replication between the CPER-derived NG-ns12.9 and the CPER-derived NG-Δns2 was not because of different unintentional mutations created by positional NG insertion.

### CPER-derived SARS-CoV-2 NG-ΔORF7a ds-circDNA produces more NG^+^ viruses than CPER-derived SARS-CoV-2 ORF3-NG ds-circDNA

**Fig 4:**
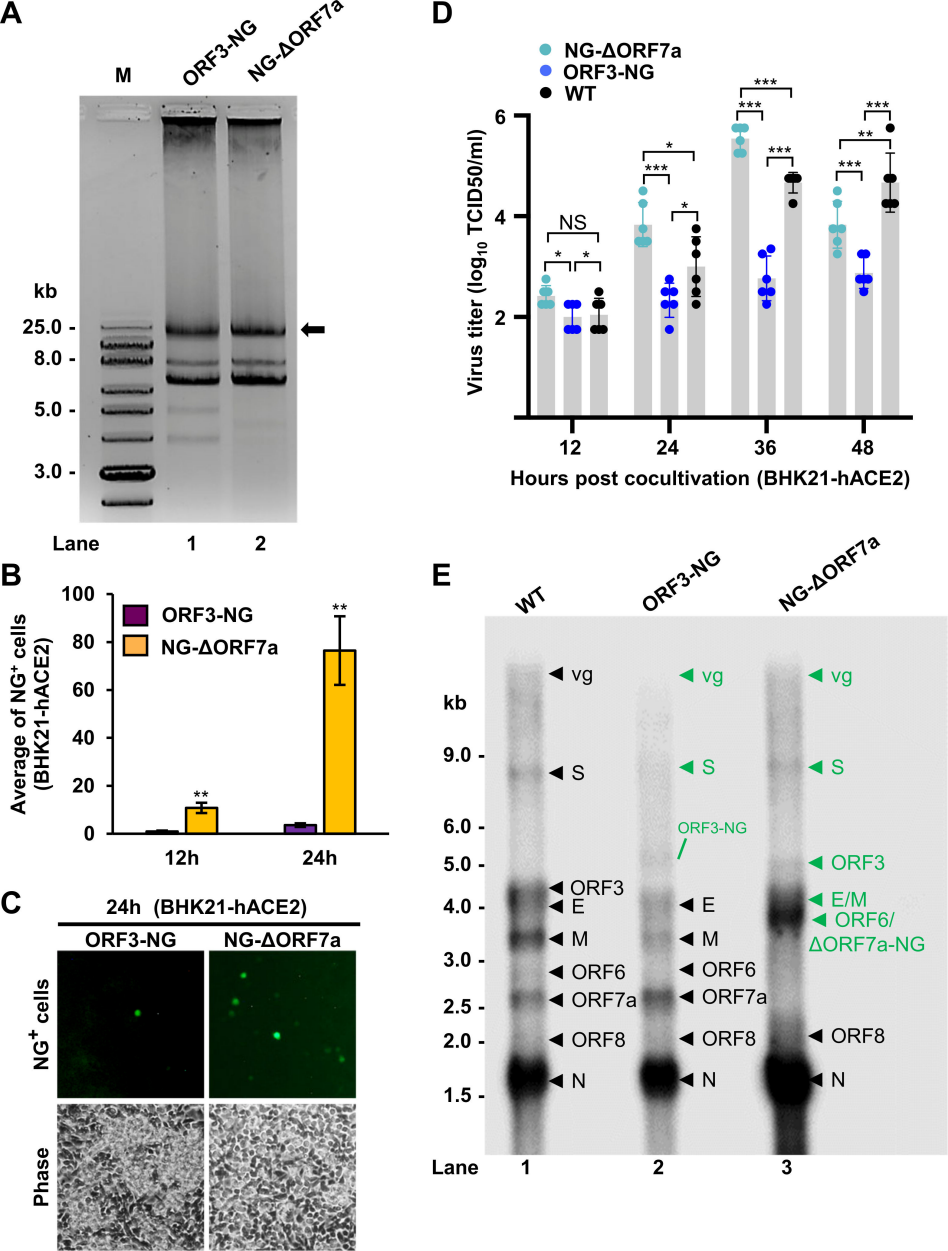
Positional NG insertion into the SARS-CoV-2 genome to determine SARS-CoV-2 replication and synthesis direction of viral sgRNAs. (**A**) Agarose gel electrophoresis of CPER-generated SARS-CoV-2 ds-circDNAs of ORF3-NG (lane 1) and NG-ΔORF7a (lane 2). See Fig. 1A for details. M, DNA kb markers. (**B and C**) Visualization and quantification of NG^+^ BHK21-hACE2 cells after co-cultivation with HEK293T cells co-transfected with CPER-derived SARS-CoV-2 ORF3-NG or NG-ΔORF7a ds-circDNA along with a SARS-CoV-2 N protein vector pCSR23. (**B**) CPER-derived NG-ΔORF7a ds-circDNA displayed a significantly higher number of NG^+^ BHK21-hACE2 cells at both 12 and 24 h than the cells co-cultivated with HEK293T cells transfected by CPER-derived ORF3-NG ds-circDNA (**, *P* < 0.01, Student’s *t*-test). (**C**) Representative images of NG^+^ BHK21-hACE2 cells 24 h after co-cultivation with HEK293T cells transfected with CPER-derived NG-ΔORF3 ds-circDNA or NG-ΔORF7a ds-circDNA. (**D**) Growth kinetics of SARS-CoV-2 WT and CPER-derived NG viruses produced from co-cultivated BHK21-hACE2 cells. The culture supernatants of infected BHK21-hACE2 cells in co-cultivation with the transfected HEK293T cells were collected at the indicated time point. After serial 10-fold dilutions, fresh BHK21-hACE2 cells in 24-well plates were infected by 100 µL of each diluent in triplicate and monitored for virus-induced cytopathic effect (CPE) for 48 h. Virus titers were calculated as TCID50 per mL and averaged from two independent experiments in triplicate. (**E**) Northern blot analysis of SARS-CoV-2 sgRNAs from BHK21-hACE2 cells co-cultivated with HEK293T cells transfected by CPER-derived ORF3-NG ds-circDNA (lane 2) or NG-ΔORF7a ds-circDNA (lane 3). Total RNA extracted from the cells 24 h post co-cultivation was analyzed by Northern blot using a ^32^P-labeled probe antisense to the SARS-CoV-2 N ORF. The RNA from the cells infected with WT SARS-CoV-2 was used as a positive control (lane 1). The sgRNAs containing an NG insert is labeled green.

The optimized CPER-based system was also applied to generate the FL SARS-CoV-2 ORF3-NG and NG-ΔORF7a (Fig. S1B and S2A; Fig. 4A, black arrow for lines 1 and 2) for co-transfection of HEK293T cells, respectively, along with a SARS-CoV-2 N protein expression vector pCSR24 (Fig. S1C) for 24 h. Subsequently, SARS-CoV-2 permissive BHK21-hACE2 cells were directly added to the monolayer of the transfected HEK293T cells for co-cultivation at the indicated time point (Fig. 4B and C). The quantification of NG^+^ cells under a fluorescent microscopy imaging for ten microscope fields at 12 h post-co-cultivation exhibited a ~10 fold higher number of NG^+^ cells from the CPER-derived NG-ΔORF7a (~10.8 NG^+^ cells/field) than the CPER-derived ORF3-NG-transfected cells (~ 0.9 NG^+^ cells/field) (Fig. 4B and C). At 24 h post co-cultivation, the difference between the NG-ΔORF7a and the ORF3-NG increased to ~20 fold, with ~76.4 NG^+^ cells/field from the NG-ΔORF7a infected cells over an average of ~3.6 NG^+^ cells/field from that of the ORF3-NG (Fig. 4B). This observation was confirmed by TCID50 titration of the collected culture supernatants for reinfection of fresh BHK21-hACE2 cells, showing higher production of infectious virus from the CPER-derived NG-ΔORF7a ds-circDNA than the ORF3-NG ds-circDNA by 12 h (*P* < 0.05) to further higher production by 24 h (*P* < 0.001) post co-cultivation (Fig. 4D).

Subsequently, we applied Northern blot using total cell RNA extracted 24 h after co-cultivation to confirm the observed difference in viral genome transcription and replication efficiency of the two CPER-derived constructs. As shown in Fig. 4E, by using an antisense ^32^P-labeled probe derived from the SARS-CoV-2 N ORF region capable of detecting viral +gRNA and all +sgRNAs, we showed a substantially higher level of viral RNAs in the cells transfected with the NG-ΔORF7a ds-circDNA than that with the ORF3-NG ds-circDNA (compare lanes 3 to 2). As expected, NG insertion and the corresponding deletion in the viral ORF7a resulted in additional 351 nt in the detected sgRNAs ORF7a, ORF6, E, M, ORF3, S, and viral gRNA (compare lanes 3 to 1, green labels at Fig. 4E; Table S2). Consistently, the NG insertion in ORF3 resulted in an increase of 708 nt in the ORF3 and S sgRNAs and the viral gRNA in the ORF3-NG ds-circDNA-transfected cells (Fig. 4E, compare lanes 2 to 1, and Table S2). Altogether with the results from hCoV-OC43, these data suggest that insertion of a NG reporter into an accessory ORF toward the viral genome 3ʹ half leads to more NG^+^ virus production than it does so by the insertion towards the viral genome 5ʹ half, most likely reflecting a graduate reduction of the read-through efficiency of individual TRS_B_-TRS_L_ cross-interactions in the discontinuous 5ʹ−3ʹ transcription template switch in the RTC.

Total cell RNA-seq and mapping indicated that both CPER-derived NG-ΔORF7a and ORF3-NG viruses contain an identical genome sequence to the original SARS-CoV-2 cDNA plasmid bearing three silent mutations at nt 26261 (C-to-U), nt 26542 (C-to-U), and nt 28853 (U-to-A) (26) (Table S4), indicating that the observed difference in viral RNA transcription and virus replication from the NG-ΔORF7a to ORF3-NG was not because of different unintentional mutations created by positional NG insertion.

### Infectious virions of hCoV-OC43 NG-Δns2 and NG-ns12.9 exhibit equal infectivity and replication capacity

**Fig 5:**
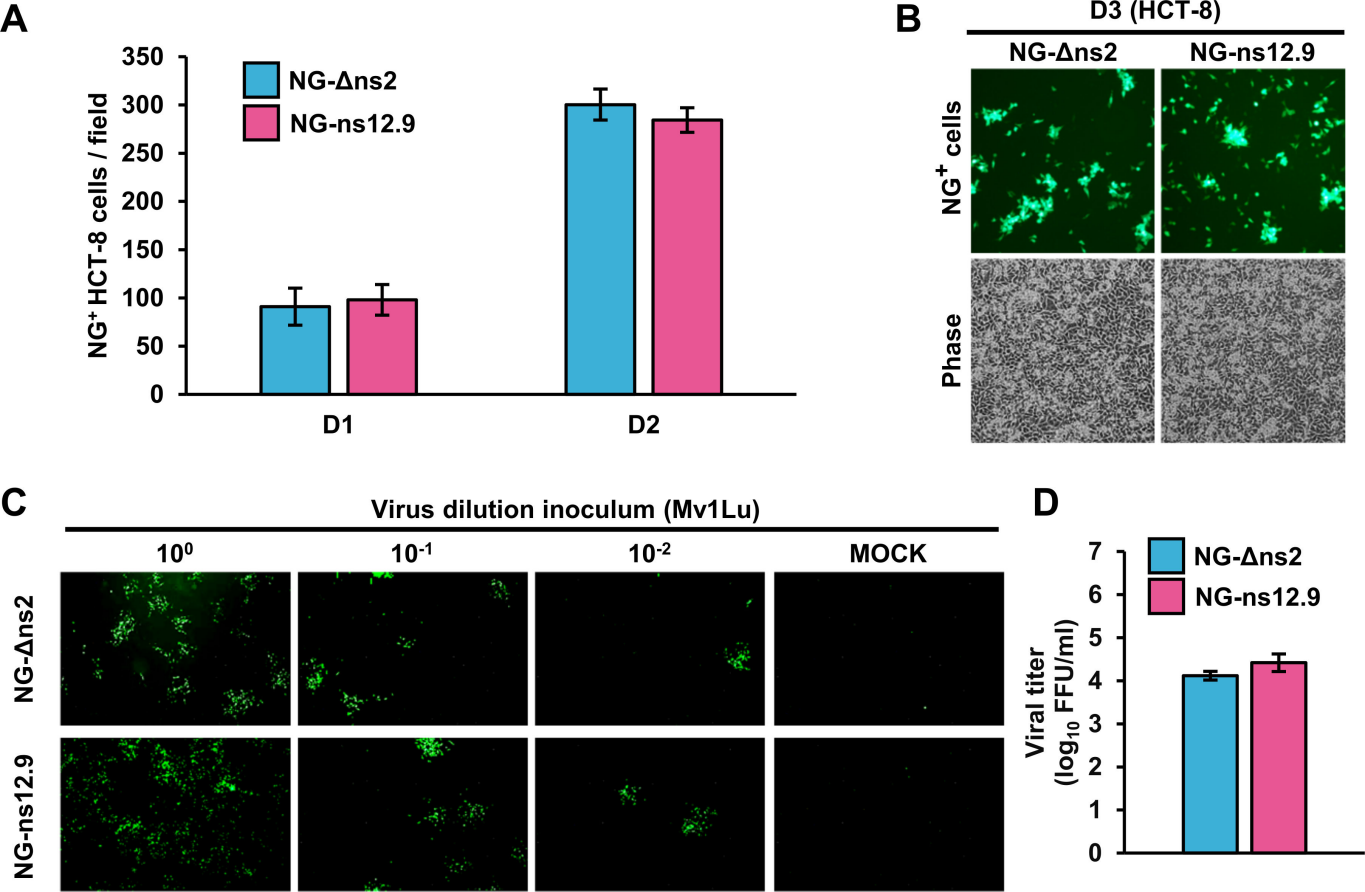
Determination of virus infectivity and replication capacity of hCoV-OC43 NG-Δns2 and NG-ns12.9 virions by *de novo* virus infection of HCT-8 and Mv1Lu cells. (**A and B**) hCoV-OC43 NG-Δns2 and NG-ns12.9 virions exhibit equal infectivity to HCT-8 cells. HCT-8 cells were infected in parallel with 0.01 MOI of hCoV-OC43 NG-Δns2 or NG-ns12.9 virions titrated, as described in Fig. 3E and F. NG^+^ HCT-8 cells were quantified from five random microscopic fields at days 1, 2, and 3 post infection (D1–D3) of the indicated virus. (**A**) No difference in NG^+^ HCT-8 cells from infection with the NG-Δns2 to NG-ns12.9 virions. (**B**) Selectively shown are one representative fluorescent image each from the HCT-8 cells infected with the NG-Δns2 or NG-ns12.9 virions at D3 post-infection. (**C and D**) hCoV-OC43 NG-Δns2 and NG-ns12.9 virions from the infected HCT-8 cell culture supernatant exhibit equal replication capacity with similar virus titers in Mv1Lu cells. The supernatant from the HCT-8 cells infected by 0.01 MOI of NG-Δns2 or NG-ns12.9 virions was collected on D3 post infection and, after serial 10-fold dilutions, 100 µL of each diluent was used to infect Mv1Lu cells, and titration of viral infectivity was carried out by the methylcellulose-overlay fluorescent foci assay (**C**). Virus titers for NG-Δns2 and NG-ns12.9 virions were calculated as fluorescent-forming units (FFU/mL) shown in a bar graph with NG-Δns2, 1.5 × 10^4^ FFU/mL and NG-ns12.9, 2.0 × 10^4^ FFU/mL (**D**).

As most of the coronavirus accessory proteins play no role in virus replication (8, 34, 35), our results from both hCoV-OC43 and SARS-CoV-2 intrigued us to further explore whether our positional NG insertions into the different viral genome regions would affect the infectivity and replication capacity of the individual CPER-derived infectious hCoV-OC43 virions. To test the replication fitness of the two NG^+^ infectious virions, we infected HCT-8 cells separately with the hCoV-OC43 NG-Δns2 and NG-ns12.9 virions titrated in Fig. 3F and justified at the same MOI 0.01 for the infection. We then monitored their replication using live fluorescent microscopy by counting NG^+^ cells in five randomly selected fluorescent fields for 3 days. We found that the infection with both hCoV-OC43 NG-Δns2 and NG-ns12.9 infectious virions replicated equally well from days 1 (D1) to 3 (D3) (Fig. 5A and B). No statistically significant difference in the number of NG^+^ cells from the NG-Δns2- to the NG-ns12.9-infected HCT-8 cells was observed at D1 (~90 NG^+^ cells/field) or D2 (~300 NG^+^ cells/field) (Fig. 5A). The Mv1Lu cell FFU assay of their cell-free virions collected on D3 cell culture supernatants further confirmed the similar kinetics of the infectivity and replication capacity of both viruses, with the NG-Δns2 viral titer reaching to 1.5 × 10^4^ FFU/mL and the NG-ns12.9 to 2.0 × 10^4^ FFU/mL (Fig. 5C and D).

Together with our total RNA-seq/viral gRNA sequencing and mapping (Table S4), the above data indicate that the insertion of an NG reporter into an accessory ORF of the virus genome either towards the 5ʹ half (NG-Δns2) or towards the 3ʹ half of the genome, once virions produced, does not induce unintentional mutation, nor the efficiency of infectivity or replication capacity of infectious virions. Whether the individual viruses exhibit a different infection index by correlation of viral gRNA copy numbers along with virus passage in HCT-8 cells to Mv1Lu infectivity remains to be carefully studied. However, the CPER strategy for the positional NG insertion does provide a simple tool to mirror the transcription efficiency of individual -sgRNAs from the viral genome in a 5ʹ to 3ʹ order of gradually reduced reading through the discontinuous template switching-mediated TRS_B_-TRS_L_ cross-interactions in the RTC (2) (Fig. 3D and 4E).

### Mapping of the hCoV-OC43 TRS_L_ and TRS_B_ in synthesis of individual sgRNAs

**Fig 6:**
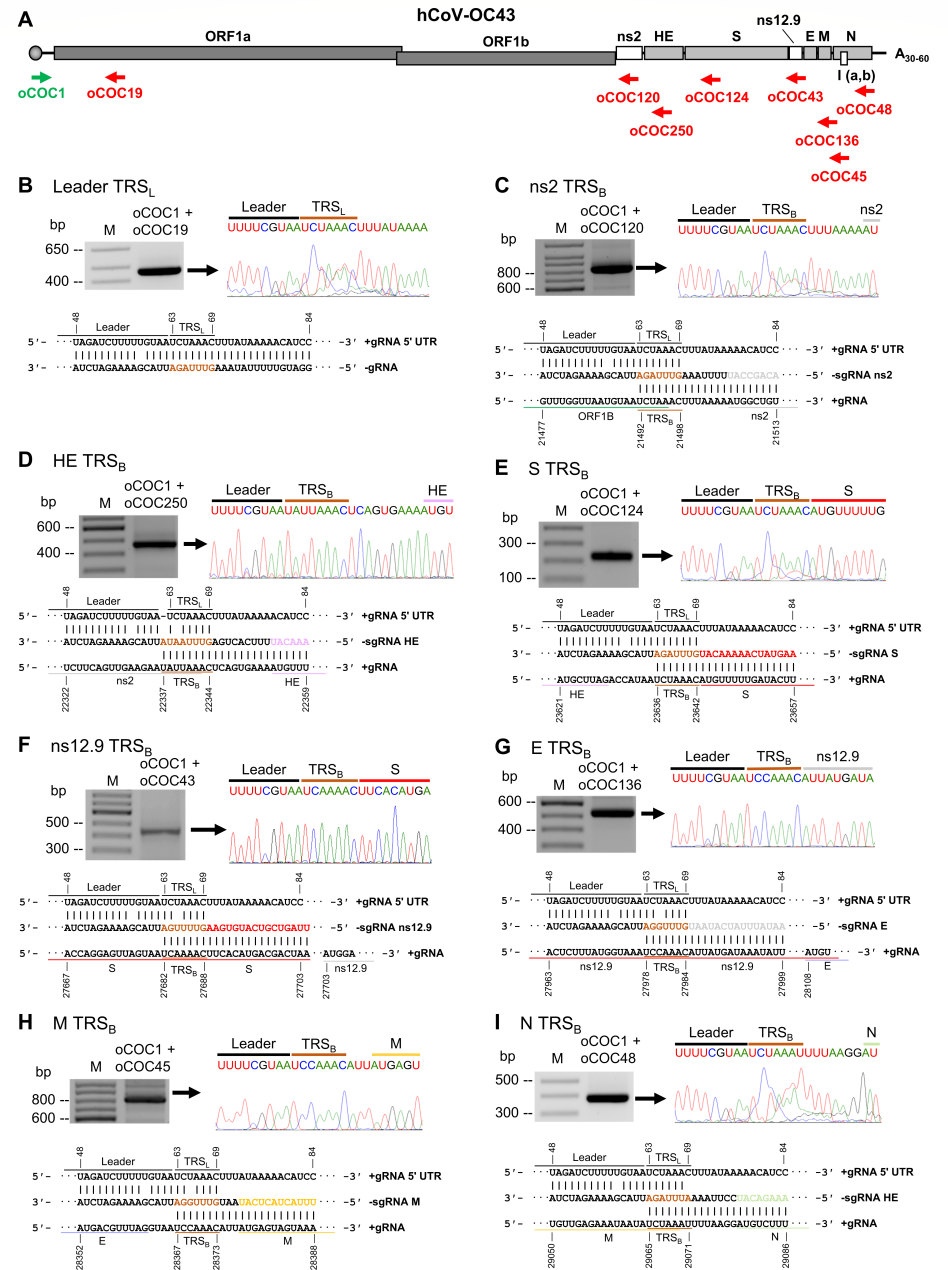
Mapping of the transcription regulatory sequence (TRS) used for synthesis of individual sgRNAs in hCoV-OC43-infected cells. (**A**) Diagram showing the localization of the forward (a green arrow) and the reverse (red arrows) primers used to map the hCoV-OC43 TRS_L_ and TRS_B_ sequence. (**B to I**) Mapping of viral TRS_L_ and TRS_B_ used for synthesis of individual hCoV-OC43 sgRNAs. RT-PCRs were carried out on total RNA extract of HCT-8 cells infected by hCoV-OC43. The amplified band corresponding to the TRS_L_ (**B**) and TRS_B_ (**C to I**) was gel-purified, sequenced, and aligned to the hCoV-OC43 reference genome (NC_006213.1).

The sequence of each TRS_B_ in regulation of hCoV-OC43 sgRNA synthesis is partially known (36) but had not been verified by other laboratories. To verify the reported TRS_L_ on the viral genome 5ʹ-end in interaction with each TRS_B_ in regulation of synthesis of individual sgRNAs (36) through a proposed template switch model (2), we performed RT-PCR on total cell RNA extracted from the WT hCoV-OC43-infected HCT-8 cells using a set of specific primers (Table S3) for detection of each sgRNA (Fig. 6A). By sequencing the amplified RT-PCR products, we confirmed that the reported TRS_L_ motif in the virus genome 5ʹ-UTR is composed of seven nucleotides, UCUAAAC (genomic position, nt 63 to 69) (Fig. 6B) (36). The same TRS_L_ sequence motif was found in the TRS_B_ of ns2 (UCUAAAC, nt 21492–21498) and S (UCUAAAC, nt 23636–23642), which might mediate the cross-interactions with the TRS_L_ motif from the viral genome 5ʹ leader for long distance looping in transcriptional template switching to synthesize individual sgRNAs containing a common 5ʹ leader (Fig. 6C and E). The TRS_B_ of ns12.9 sgRNA was mapped to the 3ʹ half of the S ORF, having a sequence motif of UCAAAAC (nt 27682–27688) differing from the TRS_L_ motif by one nucleotide (underlined) in the third position (Fig. 6F). This TRS_B_ motif is different from the reported TRS_B_ of nt12.9 (36). The E and M sgRNAs are formed by using a 7-nt motif also differing from the TRS_L_ by just one nucleotide in the third position (UCCAAAC, nt 27978–27984 and nt 28367–28373, respectively) (Fig. 6G and H). However, the TRS_B_ motif of E sgRNA was not reported (36). This TRS_B_ for synthesis of E sgRNA is positioned in the 3ʹ half ns12.9 ORF region, 123-nt upstream of the E translation initiation codon AUG. The TRS_B_ of the N sgRNA has a sequence of UCUAAAU (nt 29065–29071), as reported (36), but differs from the TRS_L_ motif by just one nucleotide in the seventh position (Fig. 6I). The mapped TRS_B_ motif for HE sgRNA in this report differs from the reported HE TRS_B_ motif (36) and is the only one containing an eight nt sequence (UAUUAAAC, nt 22337–22344) (Fig. 6D) from all other mapped 7-nt TRS_B_ motifs. All in all, we have verified and mapped all TRS_B_ motifs interacting with the TRS_L_ motif in the viral genome 5ʹ-UTR. We believe that these interactions mediate the 5ʹ leader sequence directly jumping (template switching) to the 5ʹ-end of each sgRNA to regulate translation of a viral structural or accessory protein during coronavirus infection.

### Role of the mapped ns12.9 TRS_B_ in sgRNA synthesis and virus production

After mapping all TRS_B_ motifs upstream of individual structural and accessory ORF in hCoV-OC43, we examined the function of the mapped TRS_B_ in accessory sgRNA synthesis. The accessory proteins of coronaviruses are nonessential, in general, for virus replication and infectious virus production (7, 8, 10–12, 34, 35), but hCoV-OC43 ns12.9 was recently reported as a viroporin essential for viral morphogenesis (32). However, we showed that the CPER-derived hCoV-OC43 NG-ns12.9 ds-circDNA exhibited more efficient replication and virus production than the CPER-derived hCoV-OC43 NG-Δns2 ds-circDNA (Fig. 3). The mapped TRS_B_ motif in our study (Fig. 6F) is different from the reported TRS_B_ for hCoV-OC43 ns12.9 (36). Subsequently, we examined how introduction of point mutations into our mapped ns12.9 TRS_B_ motif affects the ns12.9 sgRNA synthesis and production of infectious virus. The point mutations in the ns12.9 TRS_B_ motif were randomly introduced as silence mutations so that the coding function of individual codons in the S ORF was maintained for normal S expression in CPER-derived hCoV-OC43 NG-ns12.9 ds-circDNA.

**Fig 7:**
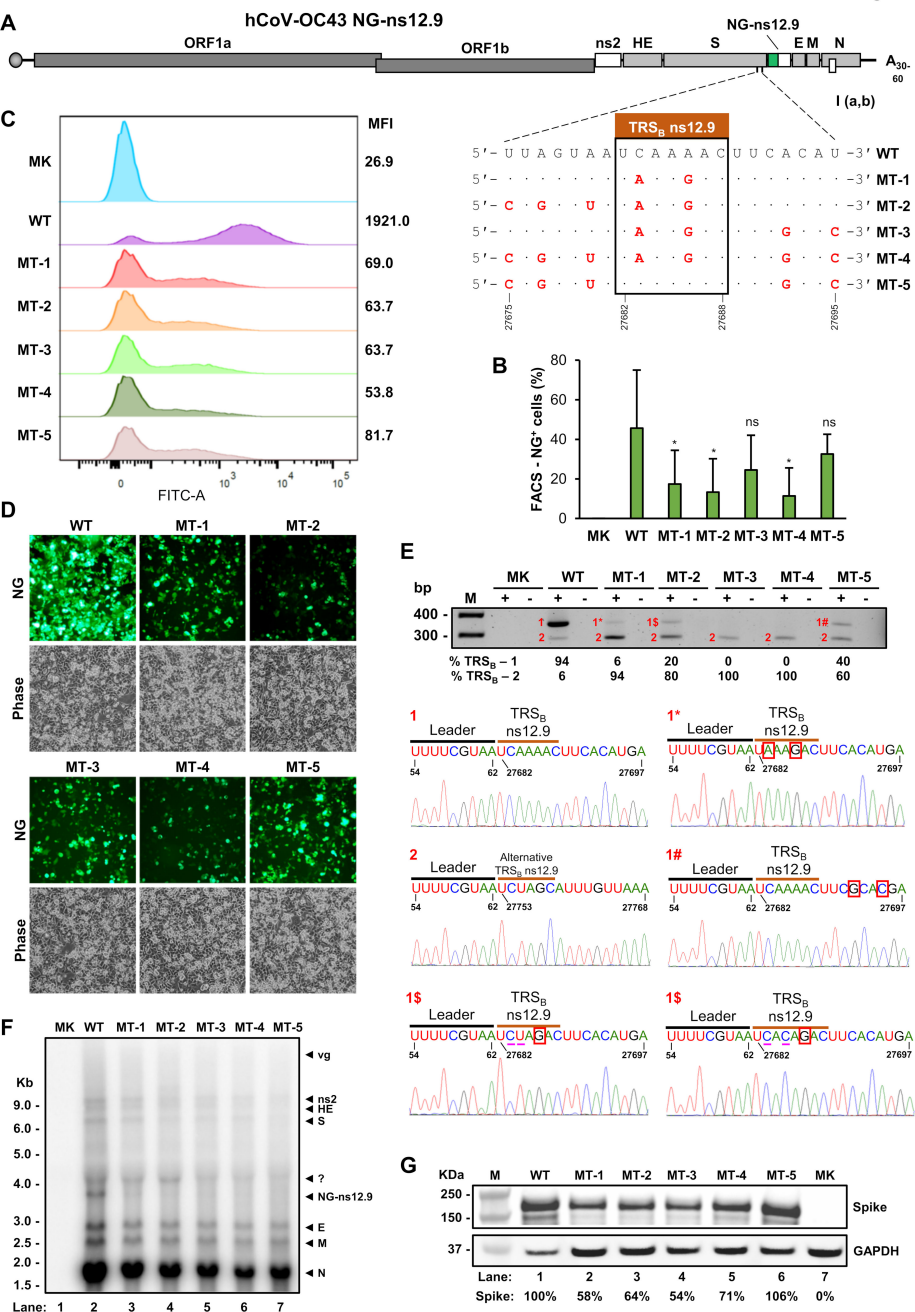
Mutation of the mapped TRS_B_ usage for synthesis of hCoV-OC43 NG-ns12.9 sgRNA activates the usage of a cryptic TRS_B_, 64 nt downstream. (**A**) Diagram showing where the silence mutations were introduced into the ns12.9 TRS_B_ core (box) and/or adjacent regions to create five mutated NG-ns12.9 ds-circDNA products (MT-1 to MT-5). (B to D) Visualization and quantification of NG^+^ HCT-8 cells after co-cultivation with HEK293T cells transfected with each CPER-derived hCoV-OC43 NG-ns12.9 ds-circDNA. Individual FL CPER products with or without TRS_B_ mutations were directly co-transfected to HEK293T cells along with a hCoV-OC43 N protein expression vector pCOC42 and co-cultivated with HCT-8 cells for 7 days post transfection before FACS analysis. (**B**) NG^+^ HCT-8 cells quantified by FACS analysis on D7 post-transfection. MK (mock infection), HCT-8 cells co-cultivated with untransfected HEK293T cells; WT, hCoV-OC43-ns12.9 with WT TRS_B_ sequence; MT-1 to MT-5 (mutant-1 to mutant-5), hCoV-OC43-ns12.9 TRS_B_ with mutated sequences of MT-1 to MT-5 shown in (**A**). Data were averaged from three independent experiments with one-tailed Student’s *t*-test. *, *P* < 0.05; ns, no statistical significance. (**C**) The median fluorescent intensity (MFI) profile is one representative FACS analysis of three experiments. (**D**) Representative microscopic images on D7 post-transfection showing NG^+^ HCT-8 cells from WT or the indicated mutants. (**E**) RT-PCR on total RNA extracted from infected HCT-8 cells on D7 post co-cultivation. Gel electropherogram shows a 347 bp (band-1) product amplified from the hCoV-OC43 NG-ns12.9 sgRNA using a WT TRS_B_. A similar band of 347 bp (1*, 1$, and 1#) was also detected from the MT-1, MT-2, and MT-5 with indicated mutations in (**A**) and their correspondent sequences. The RT-PCR products from MT-1 to MT-5 mainly generated a 276 bp product (band-2) by using an alternative TRS_B_ motif, 64 nt downstream of the WT ns12.9 TRS_B_. A nucleotide with a red box indicates the introduced mutation and with an underline indicates unexpected mutations. (**F**) Northern blot analysis of hCoV-OC43 NG-ns12.9 RNA from infected HCT-8 cells in co-cultivation with HEK293T cells transfected with the CPER-derived WT NG-ns12.9 TRS_B_ ds-circDNA (lane 2) or a mutant NG-ns12.9 TRS_B_ MT-1 to MT-5 ds-circDNA (lanes 3 to 7, respectively). Cells without transfection served as a mock infection (MK, lane 1). Total RNA extracted from the co-cultivated cells on D7 was analyzed by Northern blot using a ^32^P-labeled probe antisense to the hCoV-OC43 N ORF. The bands correspondent to each sgRNA are labeled on the right. (**G**) Western Blot analysis of total protein extracted from infected HCT-8 cells in co-cultivation with HEK293T cells transfected with the CPER-derived WT NG-ns12.9 TRS_B_ ds-circDNA (lane 1) or a mutant NG-ns12.9 TRS_B_ (MT-1 to MT-5, lanes 2 to 7) ds-circDNA to detect the presence of spike protein with an hCoV-OC43 spike-specific polyclonal antibody. The intensity of each protein band was quantified and used to calculate the relative amount of spike protein (%). The human GAPDH was used as a loading control.

Five mutants, MT-1 to MT-5, with the introduced mutations in our mapped ns12.9 TRS_B_ motif UCAAAAC or its adjacent regions, either upstream or downstream (Fig. 7A), were compared with the WT NG-ns12.9 for their replication and virus production. By transfection of HEK293T and co-cultivation with HCT-8 cells for 7 days, we quantified the number of NG^+^ cells and their fluorescent intensity by FACS (Fig. 7B and C). The WT showed ~45.6% of NG^+^ cells (Fig. 7B), with the higher NG intensity reaching a median fluorescent intensity (MFI) of 1,921.0 (Fig. 7C). As expected, we found that introduction of mutations into the ns12.9 TRS_B_ motif reduced the average number of NG^+^ cells to 17.4% (MT-1), 13.4% (MT-2), 24.6% (MT-3), 11,4% (MT-4), and 32.6% (MT-5) (Fig. 7B and D) and their MFI also dropped significantly to 69 (MT-1), 63.7 (MT-2), 63.7 (MT-3), 53.8 (MT-4), and 81.7 (MT-5) (Fig. 7C). The MT-3 had mutant TRS_B_ plus point mutations immediate downstream and showed no significant reduction of NG^+^ cells, but a remarkable reduction of MFI. The MT-5, which contains a WT TRS_B_ motif, but the mutations in the adjacent regions both upstream and downstream, also showed no significant reduction of the NG^+^ cells but a remarkable reduction of MFI when compared to the WT ns12.9 ds-circDNA (Fig. 7B through D).

Total cell RNA was isolated 7 days post co-cultivation for RT-PCR (Fig. 7E) and Northern blot (Fig. 7F) analyses. As expected, RT-PCR analysis for the WT ns12.9 TRS_B_ RNA using the primer pair described in Fig. 6A and F showed a major amplicon (347 bp) correspondent to the NG-ns12.9 sgRNA mediated by the WT TRS_B_ motif (UCAAAAC, nt 27682–27688) (Fig. 7E, band 1 and its sequence). Unexpectedly, introduction of silence mutations into the ns12.9 TRS_B_ preferentially generates a smaller RT-PCR amplicon (276 bp) (Fig. 7E, band 2), but a weak 347 bp product (Fig. 7E, bands 1*, 1$, and 1#). Sequencing this 276 bp product showed that an alternative TRS_B_ (UCUAGCA, nt 27753–27759), 64 nt downstream of the WT TRS_B_, was activated for synthesis of the ns12.9 sgRNA (Fig. 7E, band 2 and its sequence). This alternative TRS_B_ at nt 27753–27759 is not the reported TRS_B_ at nt 27771–27777 (36). Sequencing of the products 1*, 1$, and 1# showed that the 1* and 1# products had an expected sequence, respectively, from the MT-1 and MT-5, but the 1$ product from the MT-2 displayed a derived TRS_B_ motif sequence of either UCUAGAC or UCACAGAC from the introduced mutations. Together, these data suggest an important role of the mapped TRS_B_ and its surrounding sequences in guiding correct usage of the ns12.9 TRS_B_ for its sgRNA synthesis.

By Northern blot analysis, we further showed the inhibition of ns12.9 sgRNA synthesis by introduction of point mutations into the ns12.9 TRS_B_ motif (Fig. 7F, compare lane 2 to lanes 3–7). However, the profile of other sgRNA species appeared relatively normal, but a notably reduced expression with band density (Fig. 7F). Analysis of spike (S) protein production by Western blot showed a similar result (Fig. 7G). These data indicate that disruption of the ns12.9 TRS_B_ motif function by point mutations could inhibit the discontinuous transcription and protein translation. This is consistent with a previous report that ns12.9 is a viroporin responsible for virion morphogenesis and pathogenesis (32).

### The mapped TRS_B_ in hCoV-OC43 M expression is essential for sgRNA synthesis and virus production

**Fig 8:**
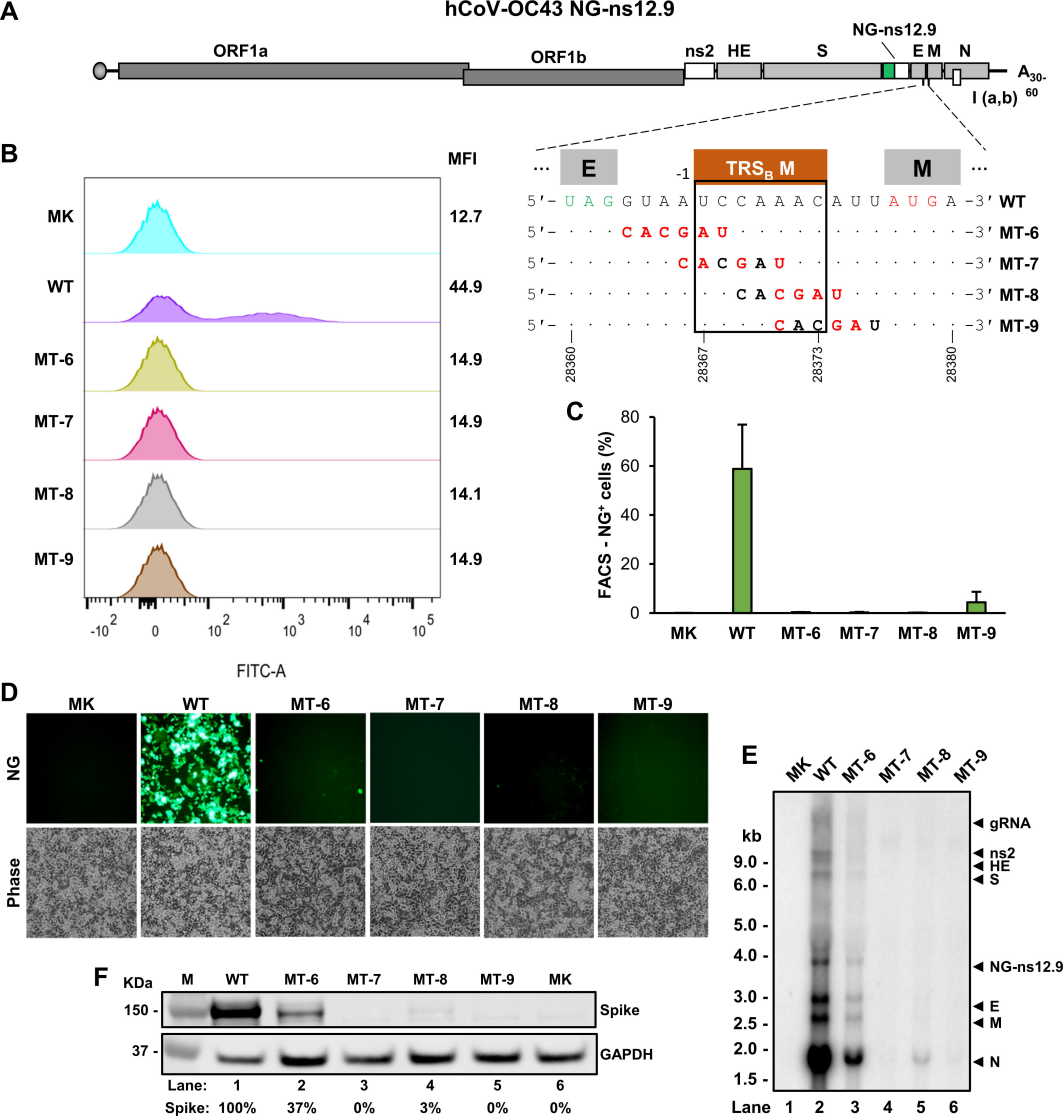
Lethal mutation of the mapped TRS_B_ usage for synthesis of the hCoV-OC43 M sgRNA from the NG-ns12.9 virus. (**A**) Diagram and nucleotide sequence show where the mutations were introduced by a 6 bp (CACGAU) linker-scanning strategy into the M TRS_B_ core (brown box) and/or adjacent regions to create four hCoV-OC43 NG-ns12.9 mutants of MT-6, MT-7, MT-8, and MT-9. (**B, C**) Visualization and quantification of the NG^+^ HCT-8 cells after co-cultivation with HEK293T cells transfected with the NG-ns12.9 ds-circDNA. Individual FL ds-circDNAs with (MT-6 to MT-9) or without (WT) mutations in the M TRS_B_ created by CPER were directly co-transfected into HEK293T cells along with a hCoV-OC43 N protein expression vector pCOC42 and then co-cultivated with HCT-8 cells for 7 days before FACS analysis of the median fluorescent intensity (MFI) of the NG^+^ HCT-8 cells (**B**). hCoV-OC43 NG-ns12.9 containing a WT M TRS_B_ sequence served as a positive control, Mock (MK) control was the HCT-8 cells co-cultivated with untransfected HEK293T cells. (**C**) Percent NG^+^ HCT-8 cells were quantified by FACS analysis from each transfection on D7 and averaged from two independent experiments. (**D**) Representative microscopic images on D7 from one of two experiments showing the NG^+^ HCT-8 cells. (**E**) Northern blot analysis of hCoV-OC43 NG-ns12.9 RNA from HCT-8 cells in co-cultivation with HEK293T cells co-transfected by CPER-derived ds-circDNAs of a WT (lane 2) or mutant (lanes 3–6) M TRS_B_ along with a hCoV-OC43 N protein expression vector pCOC42. See MK (lane 1) for details in (**B**). Total RNA extracted from the infected cells on D7 was analyzed by Northern blot using a ^32^P-labeled probe antisense to the hCoV-OC43 N ORF. The band correspondent to each sgRNA is labeled on the right. (**F**) Western blot analysis of total protein from HCT-8 cells in co-cultivation with HEK293T cells co-transfected by CPER-derived ds-circDNAs of a WT (lane 1) or MT (lanes 2–5) M TRS_B_ along with a hCoV-OC43 N protein expression vector pCOC42 to detect the expression of spike protein with an hCoV-OC43 spike-specific polyclonal antibody. MK, mock-infected cells. The band intensity was quantified to calculate the amount of spike protein (%) from each CPER-derived ds-circDNA. The human GAPDH was used as a loading control.

We further examined the mapped M TRS_B_ function in sgRNA synthesis and virus production. We introduced point mutations, by a 6 bp linker-scanning strategy, into the mapped TRS_B_ motif for the M sgRNA synthesis from the hCOV-OC43 NG-ns12.9 ds-circDNA. A 6-base linker, CACGAU, was introduced progressively from 5ʹ to 3ʹ to scan the 15 bp sequence covering the TRS_B_ motif between the ORF E and ORF M. A total of four mutants (MT-6 to MT-9, Fig. 8A) were created in the subsequent CPER reactions.

After transfection of HEK293T and co-cultivation with HCT-8 cells for 7 days, we performed FACS analysis of the transfected cells to quantify the NG expression (Fig. 8B and C). We observed that ~58.8% of the cells transfected with a WT NG-ns12.9 were NG^+^ cells (Fig. 8C) with a median fluorescent intensity (MFI) of 44.9 (Fig. 8B). All M TRS_B_ mutants displayed no or very few (< 4.5%) NG^+^ cells (Fig. 8C and D), with an MFI lower than 15 (Fig. 8B). Using a ^32^P-labeled anti-sense oligo probe from the hCoV-OC43 N ORF region capable to detect all viral +gRNA and +sgRNAs, we performed Northern blot analyses on total cell RNA collected on the D7 and showed a profile of WT virus transcription and replication (Fig. 8E), but the mutants had no or very little production of individual sgRNAs, further confirming the FACS and light microscope observations (Fig. 8B through D). Data indicate that the introduction of point mutations into the M TRS_B_ motif led to the inhibition of hCoV-OC43 virus transcription and production of infectious viruses.

Relative to the WT NG-ns12.9, the MT-6 mutant, although not lethal, displayed a remarkable sgRNA reduction (Fig. 8E) and very little spike (S) protein production (Fig. 8F). This could be because the MT-6 mutant has the 6-base linker CACGAU intruding only two positions 5’ to the M TRS_B_ motif UCCAAAC bearing an A in its −1 position (Fig. 8A). This feature makes the linker 3ʹ AU dinucleotide in the MT-6 mutant mimics the 5ʹ A/UC… of the M TRS_B_ motif by just missing one nucleotide C, consequently, resulting in a weak mutation to the M TRS_B_ motif in the MT-6 mutant. To explore this assumption, we applied the culture supernatant collected from MT-6-transfected and co-cultivated HCT-8 cells to re-infect fresh HCT-8 cells and observed outgrowth of MT-6 NG-ns12.9 virus (Fig. S4A). RT-PCR analysis of total cell RNA of HCT-8 cells at 7 days of infection gave an expected 272 bp product from the M sgRNA (Fig. S4B). Sequencing of the RT-PCR product showed a reversion of mutated MT-6 M TRS_B_ “AUCAAAC” to the WT M TRS_B_ “UCCAAAC” (Fig. S4).

## DISCUSSION

The reverse genetics technique is a common method for recombinant RNA virus production (37–39) and has been successfully applied to generate infectious clones of SARS-CoV-2 and other coronaviruses (26, 40). We have tried to use this conventional technique to generate hCoV-OC43 and hCoV-NL63 infectious clones but failed to make a complete hCoV-OC43 or hCoV-NL63 cDNA genome (data not shown) nor a complete set of their cDNA fragments due to their extremely AT-rich features (2) and unnoticed bacteria-toxic/genetic instability (41–43). The circular polymerase extension reaction or CPER, originally developed for flaviviruses (23, 44, 45), has been used to generate recombinant RNA viruses with a large genome, including coronaviruses (24, 25). In this report, we successfully adapted and optimized the published CPER protocol and efficiently constructed hCoV-OC43 and SARS-CoV-2 ds-circDNAs for production of infectious viruses, without cloning of the amplified cDNA fragment and plasmid propagation in bacteria. Using this CPER technology, we were able to insert an NG reporter into the hCoV viral genome to study TRS_B_-TRS_L_ interaction-mediated sgRNA synthesis. Subsequently, we introduced point mutations into the mapped TRS_B_ to disrupt the TRS_B_-TRS_L_ cross-interactions and the discontinuous transcription and replication. Thus, the optimized CPER technology in this report provides an alternative reliable tool for robust production of an infectious clone with any desired manipulations.

Coronaviruses synthesize their sgRNAs for translation of viral structural and accessory proteins (2, 19). The current presumption is that the -sgRNA is synthesized first from viral +gRNA by a proposed but not experimentally confirmed template switch mechanism that the 5ʹ leader sequence in the +gRNA 5ʹ-UTR could become a 5ʹ leader of each +sgRNA. This process is mediated by the cross-interactions between a TRS_B_ upstream of a given structural or accessory ORF and a distant TRS_L_ in the 5ʹ-UTR through long-range base-pairing within the RTC complex, progressing in a 5ʹ−3ʹ transcription direction from the +gRNA 3ʹ-end (2, 5, 14, 21). Consequently, these sgRNAs bearing the same 5’ leader in variable sizes appear in a magnitude order, with greatest abundance of the N sgRNA from the +gRNA 3ʹ half to the lowest for the S sgRNA located towards the +gRNA 5ʹ half, because the TRS_B_ motifs towards the viral genome 5ʹ half require more read-through steps and more time to reach during viral RNA transcription (2) (Fig. 3D, lane 3 and Fig. 4E, lane 1). Interestingly, none of these sgRNA could be included in a matured virion, with an unknown mechanism. Using the optimized CPER for positional insertion of an NG reporter into two distanced accessory ORFs, NG-ns12.9 and NG-Δns2 for hCoV-OC43, and NG-ΔORF7a and ORF3-NG for SARS-CoV-2, we were able to provide the first-hand experimental data supporting the template switch mechanism during coronaviral transcription and replication by cross-interactions between TRS_B_-TRS_L_ interaction through long-range base-pairing for viral sgRNA synthesis. We demonstrated, as expected, more production of infectious NG-viruses with the NG insertion towards the viral genome 3ʹ-half (NG-ns12.9 and NG-ΔORF7a) than that of the NG-viruses with the NG insertion towards the viral genome 5ʹ-half (NG-Δns2 and ORF3-NG), which were in parallel with the production of viral NG-sgRNAs in a magnitude abundance order (Fig. 3D and 4E).

Since cell reinfection with the same MOI of recovered hCoV-OC43 NG-ns12.9 and NG-Δns2 virions did not show any difference in virus infectivity and replication, the observed difference in NG^+^ virus production from the CPER-derived hCoV ds-circDNAs with a positional NG insertion would reflect the speed of the corresponding sgRNA synthesis in a reduced 5ʹ−3ʹ transcription progression in the discontinuous template switch. In fact, our observed results mirrored both sgRNA and thus NG-protein levels in virus transcription and gene expression. Since the matured hCoV-OC43 virions do not contain any sgRNA nor accessary proteins (in this case, NG-tagged proteins), the matured virions recovered from the infected cell culture supernatant, when adjusted to the same MOI for virion infection (Fig. 5), would lead expectably to a similar productivity, without any NG insertion-induced mutations in the virus genome (Table S4). Therefore, the positional NG insertion strategies in our study have provided an applicable tool to monitor the orientational sgRNA transcription in speed and space, thus virus replication and production.

We also verified the most of TRS_L_ and TRS_B_ 7-nt motif sequences in hCoV-OC43 reported from a previous study (36) and showed their difference mostly by one nucleotide. Other new findings from our study are as follows. (i) We mapped the TRS_B_ motif of E sgRNA, which was not identified from the previous report (36). This TRS_B_ for E sgRNA synthesis is positioned in the ns12.9 ORF region, 123 nt upstream of the E translation initiation codon AUG. (ii) We identified the TRS_B_ motif for ns12.9 sgRNA synthesis is located at the coding region of the 3ʹ S ORF, not the reported intergenic region between the S ORF and ns12.9 ORF (36). However, (iii) introduction of point mutations into our mapped TRS_B_ for the NG-ns12.9 led to a reduced synthesis of the NG-ns12.9 sgRNA and virus production because the hCoV-OC43 ns12.9 is a viroporin for virion morphogenesis and pathogenesis (32). In addition, it is possible that the silent mutations introduced into the ns12.9 TRS_B_ in the 3ʹ S ORF might affect the translation termination of S protein. (iv) The most important finding in this report is that an alternative TRS_B_ motif could be activated upon introduction of point mutations into the mapped ns12.9 TRS_B_ motif. (v) We also found that the TRS_B_ motif for the HE sgRNA has a sequence of eight nts. (vi) Introduction of mutations into the mapped TRS_B_ for structural M protein, as expected, drastically impaired the hCoV-OC43 replication and virus production, but could be rarely reverted.

**Fig 9:**
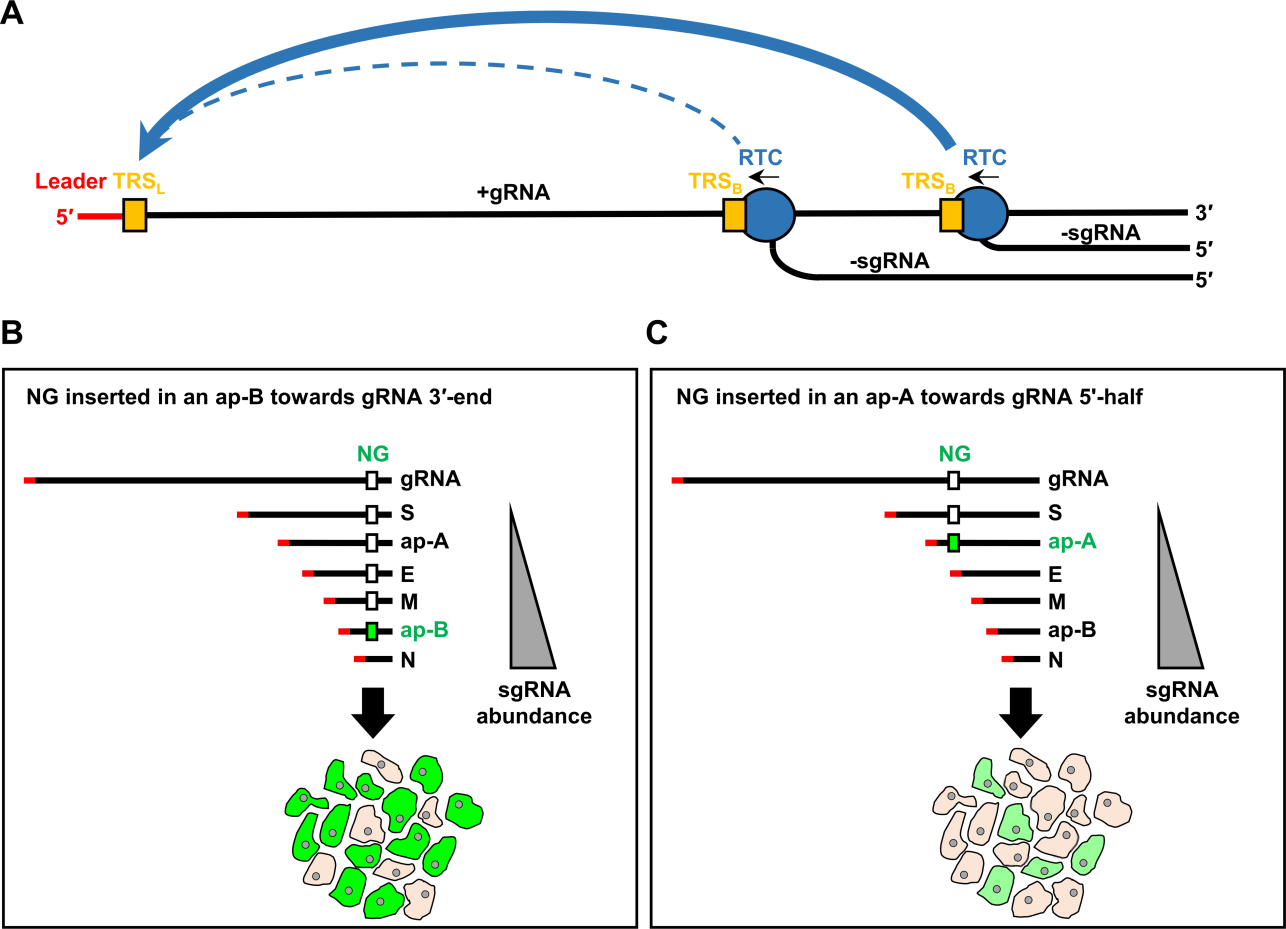
A “first-come, first-serving” model in an RTC-mediated discontinuous transcriptional template switch in long-distance of TRS_B_-TRS_L_ cross-interactions for coronavirus sgRNA synthesis. (**A**) Diagram shows -sgRNA synthesis by viral RTC from the viral +gRNA 3ʹ-end in a 5ʹ−3ʹ transcriptional direction using the viral +gRNA as a template through a template switch mechanism mediated by a long-distance TRS_B_ and TRS_L_ (orange boxes) cross-interaction in a first-come, first-serving model. Thus, a -sgRNA using a TRS_B_ closer to the +gRNA 3ʹ-end (a heavy blue arch line) will be synthesized more efficiently than those using a TRS_B_ towards the +gRNA 5ʹ half (blue dotted lines). 5’ leader, red heavy line. (**B**) As a result, insertion of an NG reporter into an accessory protein ORF (ap-B) towards the +gRNA 3ʹ-end leads to synthesis of more NG-sgRNAs and consequently more NG-tagged proteins for NG^+^ cells with strong fluorescence signals. (**C**) In contrast, insertion of the NG reporter into an accessory protein (ap-A) towards the +gRNA 5ʹ half leads to produce less NG-sgRNAs (requiring more read-through) and thus less NG-tagged protein for NG^+^ cells with weaker fluorescence signals.

In summary, we have established an efficient CPER for inserting an NG reporter into accessory ORFs in different genomic locations of hCoV-OC43 and SARS-CoV-2. We now provide experimental data to support our proposed “first-come, first serving model” for viral sgRNA synthesis, using viral +gRNA as a template through a discontinuous template switching mechanism mediated by TRS_B_-TRS_L_ long distance cross-interactions (2) (Fig. 9A). Thus, an sgRNA using a TRS_B_ near the +gRNA 3ʹ-end will be synthesized more efficiently than those using a TRS_B_ towards the +gRNA 5ʹ-half. As predicted, insertion of an NG reporter into an accessory protein ORF (ap-B) toward the gRNA 3ʹ-end would synthesize more NG-sgRNAs and consequently more NG^+^ viruses with stronger NG signals than that of an NG virus with the insertion of an NG reporter into an accessory protein ORF (ap-A) towards the gRNA 5ʹ-half (Fig. 9B and C).

## MATERIALS AND METHODS

### Coronavirus reference sequences

The nucleotide positions used in this work are derived from the reference genomes for SARS-CoV-2 (GenBank accession no. MN985325) (46) and hCoV-OC43 (GenBank accession no. NC_006213.1) (33, 36, 47) or hCoV-OC43 ATCC VR-759 (GenBank accession no. AY391777) genome sequence.

### Cell lines

The human embryonic kidney HEK293T (CRL-3216), human colorectal adenocarcinoma HCT-8 (CCL-244), monkey kidney epithelial LLC-MK2 (CCL-7) and American mink lung epithelial Mv1Lu (CCL-64) cells were obtained from ATCC. Stable transfection with hACE2 receptor plasmid was also used to generate hamster kidney fibroblast BHK21-hACE2 cells (48). All cell lines were maintained in complete Dulbecco’s modified Eagle medium (DMEM, Thermo Fisher Scientific) supplemented with 10% fetal bovine serum (FBS, Cytiva) and 1× penicillin-streptomycin-glutamine (PSG, Thermo Fisher Scientific) at 37°C in 5% CO_2_ atmosphere.

### Virus stocks

The hCoV-OC43 (VR-1558 originated from VR-759) virus stock was obtained from ATCC. hCoV-OC43 was propagated in HCT-8. SARS-CoV-2 from BEI Resources was propagated in BHK21-hACE2 cells. The cells were infected by 1 h virus adsorption at 37°C. Unbound virus was washed away, and cells were incubated with DMEM (2% FBS) at 33°C for 7 days or until appearance of the cytopathic effect (CPE). The culture supernatant was collected and frozen at −80°C in 1 mL aliquots containing 10% DMSO. The infected cells were lysed in TriPure Isolation Reagent (Roche) for total RNA extraction or in 2× LDS protein sample buffer containing 5% mercaptoethanol for protein detection by Western blot.

### Preparation of hCoV-OC43 and SARS-CoV-2 specific linkers for CPER

We obtained the linker plasmid and sequence from Alberto A. Amarilla and Alexander A. Khromykh (25) and further verified in our laboratory. This linker in size of 1,119 bp is a central component for efficient CPER and contains a 30 bp stretch of adenosine sequence, a 113 bp hepatitis delta virus ribozyme (HDVr), an SV40 poly-A (pA) signal, and a CMV IE promoter (Fig. S1A). The linker was amplified by PCR to have 20 bp nucleotides from the viral genome 3ʹ-UTR on the linker 5ʹ-end and 37 bp nucleotides from the viral genome 5ʹ-UTR on the linker 3ʹ-end. This allows the linker to circle the virus genome from its 3ʹ-end to the 5ʹ-end in a CPER, enabling the CMV IE promoter upstream of the viral 5ʹ-UTR for viral transcription and the HDVr downstream of the 30-nt stretch of adenosines to produce homogenous viral RNAs with the same 3ʹ-end by ribozyme-mediated self-cleavage (23, 25, 44, 45, 49).

### Preparation of cDNA fragments from hCoV-OC43 and SARS-CoV-2

As diagrammed in Fig. S2Aand B, and Table S1, we amplified by PCR using high-fidelity Platinum SuperFi II DNA Polymerase seven overlapping cDNA fragments (F1 to F7), respectively, from FL SARS-CoV-2 and hCoV-OC43 genomic cDNAs (Fig. S1B, steps 1 and 2, and Table S1), with a 20–40 nt overlapping sequence from each fragment. The fragment positions from individual coronavirus genomes and oligos used for the amplification are summarized Tables S1 and S3. By annealing the overlapped cDNA fragments and the viral specific linker (Fig. S1B, step 3), the semicircular cDNA could be formed through base-pairing of the overlapped sequences from one fragment to another, and each fragment can be served as a template and also as a primer to fill the gap between two fragments by PrimerSTAR GXL DNA polymerase (Takara) (Fig. S1B, step 4), finally resulting in generation of ds-circDNAs (Fig. S1B, step 5).

We obtained infectious SARS-CoV-2 cDNA clones from Dr. Pei-Young Shi (26) originated from the first US-reported SARS-CoV-2 strain (2019-nCoV/USA_WA1/2020) and made a SARS-CoV-2-specific linker. Two (F6 and F7) of seven fragments derived from this SARS-CoV-2 cDNA were used for insertion of an NG reporter (22). The F6 had a NG fused downstream in frame with accessory ORF3 (ORF3-NG) and the F7 had a NG replacement of accessory ORF7a (NG-ΔORF7a) (Fig. 1A; Fig. S2A).

Seven (F1 to F7) cDNA fragments (Fig. S2B and Table S1) covering the entire hCoV-OC43 genome were amplified from an FL hCoV-OC43 cDNA obtained from hCoV-OC43-infected HCT-8 cells (Fig. S2B), and a hCoV-OC43 specific linker was made according to the hCoV-OC43 genome sequence. The F5 fragment from hCoV-OC43 cDNA had an NG replacement of the N-terminal accessory ORF2 (NG-Δns2, Fig. 1A) or an NG insertion upstream of the ORF2 (NG-ns2) (Fig. S2B). The NG-ns2 had a picornavirus 2A ribosomal skipping sequence (T2A, black box) insertion between the NG and the ns2 (Fig. S2B), and F7 from hCoV-OC43 cDNA had an NG insertion upstream of the accessory ORF12.9 (NG-ns12.9), separating again by a T2A sequence (50)(black box in Fig. S2B).

hCoV-OC43 fragment 2 (from nt 3646 to nt 8298) was amplified by PCR with an oligo pair of oCOC3 and oCOC4 using hCoV-OC43 cDNA as a template. The final PCR product was column-purified and cloned at the pCR-XL-2 TOPO vector (Thermo Fisher Scientific). The BsaI restriction sites were introduced by the oCOC3 and oCOC4. Plasmid pCOC4 obtained was verified by sequencing and used to determine hCoV-OC43 copy number.

### Optimizing CPER for production of coronavirus infectious ds-circDNAs

The CPER (23, 24, 25) was performed with some modifications using 7 overlapping cDNA fragments (Fig. S2A and B) plus a coronavirus-specific linker using high-fidelity Platinum SuperFi II DNA Polymerase (Thermo Fisher Scientific). We determined the optimal fragment concentration for individual viral CPER by using 0.01, 0.05, and 0.1 pM of individual gel-purified cDNA fragments and a virus-specific linker (Fig. 1B) in a 50 µL reaction containing 1× GXL buffer, 200 µM dNTP mix, and 2 µL of PrimerSTAR GXL DNA polymerase (Takara). The CPER cycling conditions are as follows: initial denaturation at 98°C for 30 seconds; 12 cycles of denaturation at 98°C for 10 seconds, annealing a 55°C for 20 seconds and extension at 68°C for 10 minutes; and final extension of 68°C for 10 minutes. The efficacy of CPER was determined by electrophoreses in a 0.8% agarose gel, and final product size was estimated based on ExcelBand XL 25 kb DNA ladder (SMOBIO).

### Cell transfection and co-cultivation of CPER-derived viral ds-circDNAs

CPER-derived FL ds-circDNAs of SARS-CoV-2 or hCoV-OC43, without gel-purification, were used to transfect HEK293T cells in two wells of a 6-well plate (each well having 0.25 × 10^6^ cells seeded 24 h before transfection) using Lipofectamine LTX PLUS Reagent (Thermo Fisher Scientific). Prior to transfection, the medium was replaced with fresh DMEM containing 2% FBS, and each well of the cells was co-transfected with 25 µL of the CPER products mixed with 1 µg of virus-specific N expression plasmid (pCSR24 for SARS-CoV-2, pCOC42 for hCoV-OC43). The transfected cells were incubated at 37°C for 24 h before addition of 1 × 10^6^ cells per well of BHK21-hACE2 for SARS-CoV-2 or of HCT-8 for hCoV-OC43 in DMEM with 10% FBS for another 48 h incubation at 33°C. The co-cultured cells were then passed into a T75-flask and cultivated in DMEM with 10% FBS at 33°C for the indicated time point.

Virus growth in CPER-transfected cells was monitored daily for NG expression by direct fluorescent microscopy for a period of 2–5 days, and the number of NG^+^ cells was determined in ten randomly selected microscopy fields. Alternatively, the number of NG^+^ cells was quantified by flow cytometry (FACS) on LSR II system (BD Biosciences) and the data analyzed with FlowJo Software. Finally, the culture supernatant containing infectious virions was collected and stored in 1 mL aliquots containing 10% DMSO at −80°C. The remaining cells in half were lysed in 5 mL of TriPure Isolation Reagent (Roche) for total RNA isolation or lysed in 2 X SDS buffer for protein assays.

### Plaque assay by using LLC-MK2 and Mv1Lu cells

LLC-MK2 and Mv1Lu cells were used for plaque assays using serial 10-fold dilutions of WT hCoV-OC43 virus-containing cell culture supernatants from infected HCT-8 cells. In brief, 0.5 × 10^6^ Mv1Lu cells per well were seeded in a 24-well plate for 24 h and then infected in duplicate with 100 µL of a tenfold-dilution of the HCT-8 cell culture supernatant in serum-free DMEM. Following 1 h absorption at 37°C, the infected cells were rinsed with 1 mL 1 x PBS, overlayed with 1 mL of semisolid DMEM-methylcellulose media (1 x DMEM with 10% FBS, 0.5% methylcellulose, 0.25% NaHCO_3_, and 1% PSG), and then incubated at 33°C for 8 days before fixation with 3.7% formaldehyde for 30 minutes and staining for 5 min with 1% crystal violet. The visible plaques per well were counted to calculate viral titters in plaque-forming units (PFU/mL).

Alternatively, number of fluorescent plaques per well in an NG-containing coronavirus infection could be determined by fluorescent microscopy as fluorescent-forming units (FFU). The virus titer per mL (FFU/mL) could be further calculated.

### TCID50 assay

The culture supernatants of infected BHK21-ACE2 cells in co-cultivation were collected at the indicated time. After serial 10-fold dilutions, fresh BHK21-hACE2 cells in 24-well plates were infected by 100 µL of each diluent in triplicate and monitored for virus-induced cytopathic effect (CPE) for 48 h. Virus titers are calculated as TCID50/mL.

### Northern blot

Total RNA (5–30 µg) was separated by 1% formaldehyde-denaturing agarose gel electrophoresis in 1× MOPS buffer together with an RNA Millennium Marker (Thermo Fisher Scientific), transferred to a nylon membrane, and probed with an antisense ^32^P-labeled oligo specific to a coronavirus N ORF region for detection of all viral transcripts (Table S3). The ribosomal RNA by ethidium bromide (EtBr) staining was used as a loading control.

### Western blot

Total cell lysates were resolved on a 4%–12% Bis–Tris NuPAGE gel (Thermo Fisher Scientific), transferred to a nitrocellulose membrane, and blotted with a rabbit anti-hCoV-OC43 Spike (S) protein antibody (E4U6P, #16435 Cell Signaling Technology) or mouse anti-GAPDH (D4C6R, #97166 Cell Signaling Technology) as a loading control. After blotting with a secondary anti-rabbit antibody, the immunoreactive proteins were detected with SuperSignal West Pico PLUS Chemiluminescent Substrate (Thermo Fisher Scientific), and the signal was captured by ChemiDoc Touch imaging system (Bio-Rad).

### TRS mapping and mutational analysis

The cDNA from infected cell total RNA was used to amplify the hCoV-OC43 leader-body junction by Platinum SuperFi II DNA Polymerase (Thermo Fisher Scientific) using a forward leader primer (oCOC1) in combination with an individual sgRNA-specific reverse primer (Fig. 6A; Table S3). The amplified products in the predicted size were gel-purified and Sanger-sequenced. TRS_B_ mutant-containing DNA fragments were generated by overlapping PCR and used by CPER to construct the expected hCoV-OC43 NG-ns12.9 ds-circDNA for the subsequent assays.

### hCoV-OC43 nps3 TaqMan assay and calculation of viral genome copy numbers

Forward primer 5ʹ-TTCCATTCAGGATGTGGGTTT-3ʹ (nt 6971–6991) and a reverse primer 5ʹ-AAATGCTCTCCTATCAGCTTCAT-3ʹ (nt 7087–7109), along with a probe 5ʹ−6-FAM/TTGCATGTC/ZEN/AGTTCTGCTTGGCAG/3ʹ-IABkFQ (nt 7018–7041), were used for the RT-qPCR to quantify the total viral genomic RNA isolated from infected HCT-8 cells. Copy number of the virus gRNA was calculated using a standard curve generated using pCOC4 plasmid containing an hCoV-OC43 genome region from nt 3646 to 8298.

### Total RNA-seq and viral gRNA sequencing

The A549-ACE2 cells were infected with SARS-CoV-2 WT, ORF3-NG, or NG-ΔORF7a virus with 0.05 MOI. The total cell RNA was extracted at 48 h postinfection using TriPure Reagent. HCT-8 cells were infected with 100 µL of hCoV-OC43 WT, NG-Δns2, and NG-ns12.9 virus inoculum. The viral RNA was isolated from 1 mL of the infected HCT-8 cell culture supernatant harvested 6 days post infection using the Zymo Quick-RNA Viral Kit.

All RNA samples were converted to sequence libraries using the TruSeq Stranded Total RNA Kit and sequenced by Illumina MiSeq platform. Sequencing adapters from R1 and R2 FASTQ files were trimmed, and low-quality reads were removed. The filtered RNA reads from each sample were mapped to chimeric hg38-SARS-CoV-2 (GenBank accession no. MN985325) or hg38-hCoV-OC43 (GenBank accession no. AY391777) reference genomes. The consensus sequences of individual viruses were extracted using IGV and aligned with the reference genome using Clustal Omega. The observed mutations on protein-coding potential were determined based on annotated ORF.

### Statistical analysis

For comparison between two groups, we performed a two-tail Student’s *t*-test. For comparison between multiple groups, we performed one-tail Student’s *t*-test or ANOVA test with Bonferroni correction. *, *P* < 0.05; **, *P* < 0.01; ***, *P* < 0.001; ns, no significance.

## Data Availability

Total cell RNA-seq and viral gRNA sequencing data of hCoV-OC43-infected HCT-8 cells are available upon request.

## SUPPLEMENTAL MATERIAL

Supplemental material jvi.01438-25-s0001.pdf Figures S1 to S4; Tables S1, S2, and S4.

Table S3 jvi.01438-25-s0002.xlsx Oligos used in this report and their sequence positions in each virus genome.

ASM does not own the copyrights to Supplemental Material that may be linked to, or accessed through, an article. The authors have granted ASM a non-exclusive, world-wide license to publish the Supplemental Material files. Please contact the corresponding author directly for reuse.
