# Supplementary material for: Discontinuous template switching generates coronavirus subgenomic RNAs from the 3ʹ viral genome end by 5ʹ to 3ʹ transcription
Source: J Virol. 2025 Sep 25;99(10):e01438-25. doi: 10.1128/jvi.01438-25 (PMC12548397; doi:10.1128/jvi.01438-25)
Supplement: Supplemental material — Figures S1 to S4; Tables S1, S2, and S4. [file jvi.01438-25-s0001.pdf]

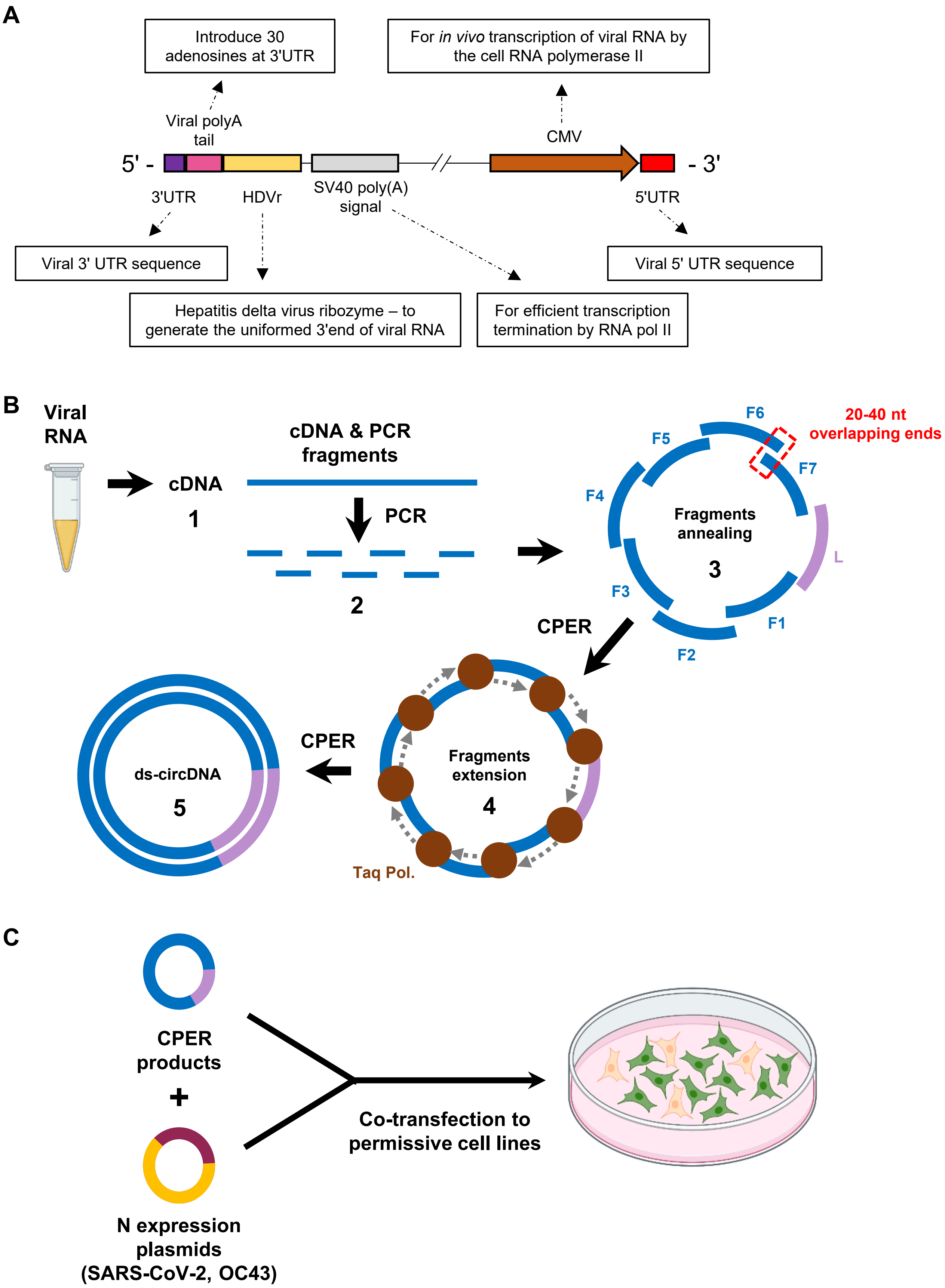
A
Introduce 30 adenosines at 3'UTR
For *in vivo* transcription of viral RNA by the cell RNA polymerase II
Viral polyA tail
CMV
5' -
- 3'
3'UTR
HDVr
SV40 poly(A) signal
5'UTR
Viral 3' UTR sequence
Viral 5' UTR sequence
Hepatitis delta virus ribozyme – to generate the uniformed 3'end of viral RNA
For efficient transcription termination by RNA pol II
B
Viral RNA
cDNA
1
cDNA & PCR fragments
PCR
2
F6
20-40 nt overlapping ends
F5
F7
F4
Fragments annealing
3
L
F3
F1
F2
CPER
CPER
ds-circDNA
5
Fragments extension
4
Taq Pol.
C
CPER products
+
N expression plasmids (SARS-CoV-2, OC43)
Co-transfection to permissive cell lines

Fig S1: Diagrams and workflow of CPER technology for construction of an infectious coronavirus clone.
(**A**) Diagram of the CPER linker, which is 1119-bp long and plays a central role in CPER, is composed of several elements essential for an efficient in vivo transcription and production of an infectious coronavirus clone. The linker 5'-end contains a 20-bp sequence (purple box) overlapping with the coronaviral 3'-UTR end, and the linker 3'-end has a 37-bp sequence (red box) overlapping with the coronaviral 5'-UTR end. These two regions of the linker, respectively, base-pair with the last and first viral cDNA fragments for CPER circularization (see panel B). The linker also contains 30 adenosines as a viral polyA-tail (pink box) at the coronaviral 3'-UTR, a hepatitis delta virus ribozyme (HDVr-yellow box), a SV40 poly(A) signal (grey box), and a CMV promoter (brown filled arrow) immediately upstream of the coronaviral 5'-UTR for transcription of the viral RNA by the cellular RNA pol II. The SV40 poly(A) signal is for efficient transcription termination and the HDVr for self-cleavage of newly synthesized coronaviral RNA transcripts in the cells to secure the production of homogenous coronaviral RNA with the same 3'-end (49). (**B**) A workflow chart of CPER strategy for construction of FL coronaviral ds-circDNAs. A FL cDNA synthetized from each viral genomic RNA [step 1] was used as a template to amplify 5-7 cDNA fragments [steps 2 and 3] (blue F1 to F7). Each cDNA fragment contains an overlapping end of 20-40 bp for base-pairing in a right orientation and with a linker fragment (pink) [step 3], forming semi-circular cDNA by annealing. During the CPER reaction, each cDNA fragment and the linker partially base-paired serves as a template and as a primer for extension to fill the gap (dashed grey arrows) between two fragments by DNA polymerase (brown circles) [step 4] and thus form the expected ds-circDNA [step 5].
(**C**) The resulting ds-circDNAs were used for co-transfection of permissive cells with a virus-specific N protein expression plasmid. Part of the figure was designed with BioRender.

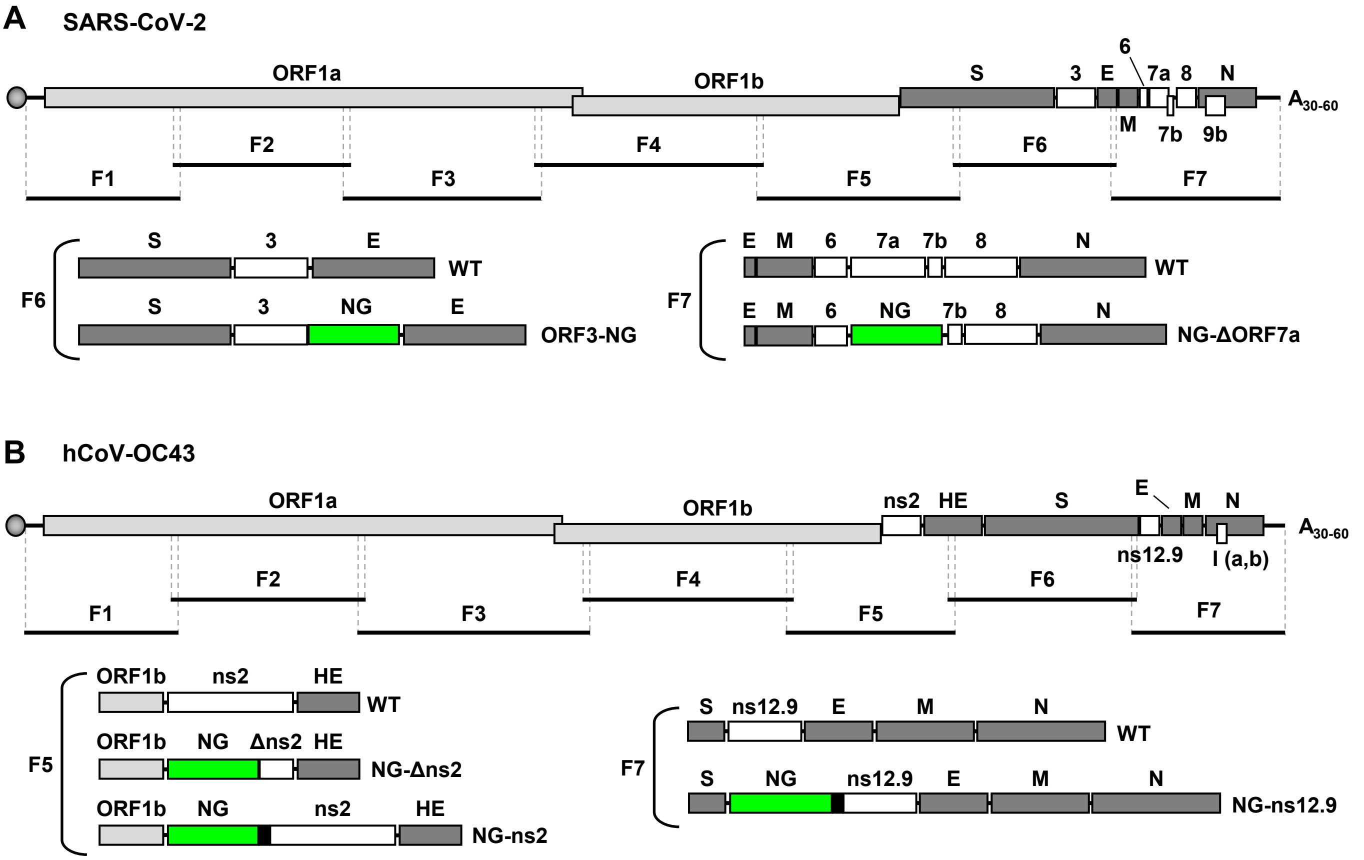

Fig S2. Generation of viral cDNA fragments with overlapped sequences.

Diagrams of the SARS-CoV-2 and hCoV-OC43 genomes with the ORFs coding for non-structural (light gray box), structural (dark grey box) and accessory proteins (white box). To generate an infectious clone by CPER, the FL SARS-CoV-2 **(A)** or hCoV-OC43 **(B)** cDNA was amplified into seven cDNA fragments (F1 to F7). Each cDNA fragment overlaps by 40 nucleotides with its two adjacent fragments. To introduce a mNeonGreen (NG, green boxes) reporter into the indicated accessory protein ORF, the corresponding cDNA fragment was manipulated to have the NG replace or fuse with the selected accessory ORF. **(A)** For SARS-CoV-2, the NG was fused to ORF3 as an ORF3-NG in F6. On the other hand, ORF7a in F7 was replaced by NG as an NG-ΔORF7a. **(B)** For hCoV-OC43, the NG was fused to a partially deleted ns2 in F5 as an NG-Δns2 or to a FL ns2 as an NG-ns2. However, the NG-ns2 has a picornavirus 2A ribosomal skipping sequence (T2A, black box) between the NG and the ns2. Moreover, the NG was fused to ns12.9 in F7 as an NG-ns12.9.

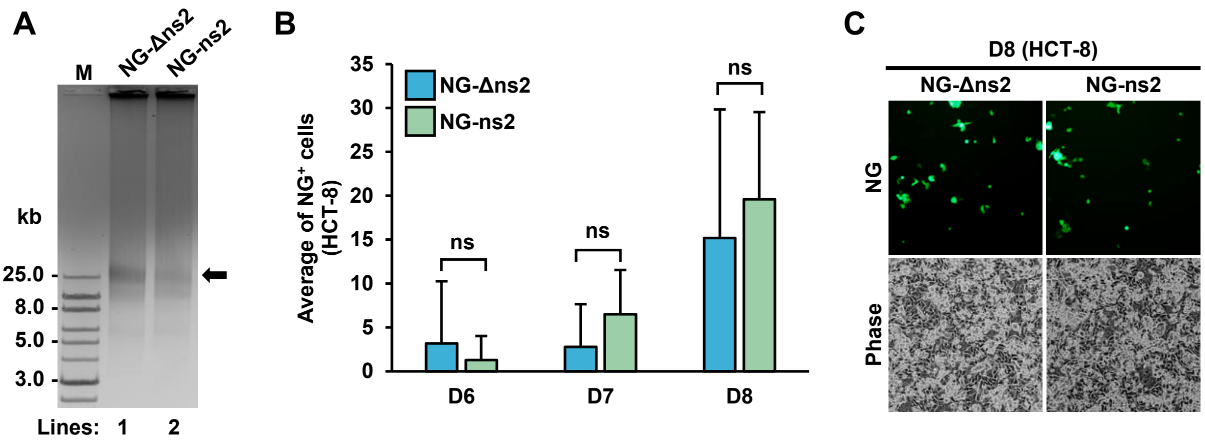

Fig S3: ORF ns2 is not essential for hCoV-OC43 replication in HCT-8 cells.

(A) Agarose gel (0.8%) electrophoresis of the FL (~30 kb) ds-circDNA product (black arrow) constructed by the CPER for hCoV-OC43 NG-Δns2 (lane 1) and hCoV-OC43 NG-ns2 (lane 2). **(B-C)** HEK293T cells were co-transfected with a FL NG-NG-Δns2 ds-circDNA or NG-ns2 ds-circDNA along with an hCoV-OC43 N expression vector pCOC42 for 24 h and then co-cultivated with HCT-8 cells for 8 days. Numbers of the NG^+ cells were quantified and averaged from 10 random microscopy fields on days 6, 7, and 8 (D6-D8). No statistical (ns) difference was found between the two constructs using Student's *t*-test. **(C)** One representative microscopic field is shown for NG^+ HCT-8 cells infected with hCoV-OC43 NG-Δns2 or NG-ns12.9 virus at D8.

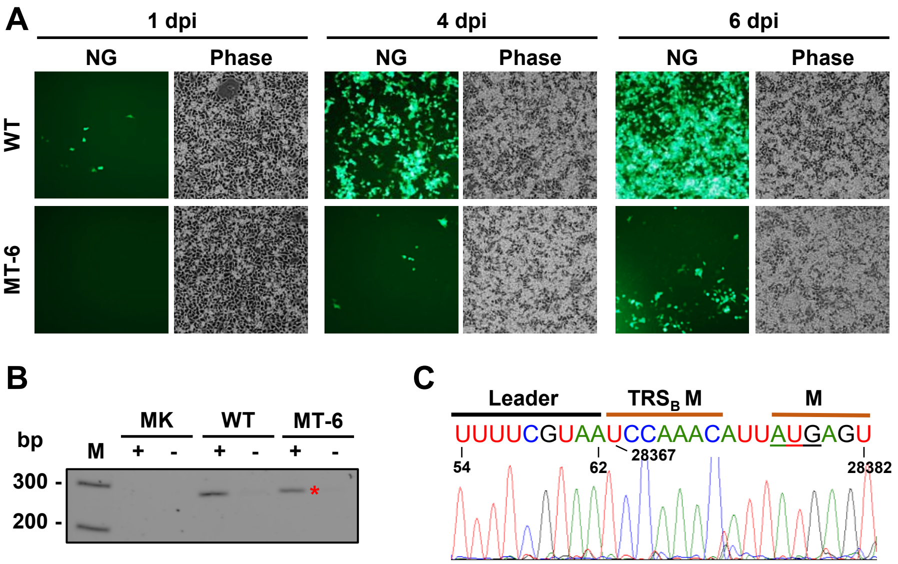

Fig S4. Outgrowth of a TRS_B reverted MT-6.

(**A**) The representative images of HCT-8 cells infected with hCoV-OC43 WT or MT-6 NG-ns12.9 virus collected at 1, 4 and 6 days of post infection (dpi). The NG-positive cells are in green. **(B)** The agarose gel showing a 272-bp RT-PCR product of the M sgRNA obtained from total cell RNA of HCT-8 cells infected at 7 dpi with WT or MT-6 virus. (**C**) The chromatograph of Sanger sequencing of the RT-PCR product amplified from the MT-6 M sgRNA in (B), showing a reversion of MT-6 M TRS_B “AUCAAAC” to the WT M TRS_B “UCCAAAC”.

Table S1. Human coronaviruses cDNA fragments amplification for CPER

SARS-Cov-2 (Ref #: MN985325)

Fragment	Start	End	Oligo F	Oligo B	Template	Size
Linker	1/37	29851/29870	oCSR99	oCSR100	Linker plasmid	1119 bp
F1	17	3639	oCSR113	oCSR102	pUC-F1 Plasmid	3623 bp
F2	3600	7524	oCSR101	oCSR104	pUC-F2 Plasmid	3925 bp
F3	7485	12004	oCSR103	oCSR106	pUC-F3 Plasmid	4520 bp
F4	11956	17611	oCSR105	oCSR108	pUC-F4 Plasmid	5647 bp
F5	17572	22068	oCSR107	oCSR110	pUC-F5 Plasmid	4497 bp
F6	22029	26352	oCSR109	oCSR112	pUC-F6 Plasmid	4324 bp
F6-NG (ORF3-NG)	22029/26217	26221/26352	oCSR109	oCSR112	cDNA	5032 bp
F7	26313	29870	oCSR111	oCSR114	pUC-F7 Plasmid	3558 bp
F7-NG (NG-ΔORF7a)	26313/27393	27762/29870	oCSR111	oCSR114	pUC-F7NG Plasmid	3909 bp

hCoV-OC43 (Ref #: NC_006213.1)

Fragment	Start	End	Oligo F	Oligo B	Template	Size
Linker	1/37	30694/30713	oCOC197	oCOC198	Linker plasmid	1119 bp
F1	17	3665	oCOC211	oCOC200	cDNA	3649 bp
F2	3626	8318	oCOC199	oCOC202	cDNA	4693 bp
F3	8279	13665	oCOC201	oCOC204	cDNA	5387 bp
F4	13626	19170	oCOC203	oCOC206	cDNA	5545 bp
F5	19131	22707	oCOC205	oCOC208	cDNA	3577 bp
F5-NG (NG-Δns2)	19131/21505	22223/22707	oCOC205	oCOC208	pCOC2	3571 bp
F5-NG (NG-ns2)	19131/21505	21506/22707	oCOC205	oCOC208	cDNA	4339 bp
F6	22668	27579	oCOC207	oCOC210	cDNA	4912 bp
F7	27540	30713	oCOC209	oCOC212	cDNA	3174 bp
F7-NG (NG-ns12.9)	27540/27791	27792/30713	oCOC209	oCOC212	cDNA	3936 bp

Table S2. Viral gRNA and sgRNAs sizes from infected cells with a wild type virus or CPER-derived ds-circDNA transfection

SARS-Cov-2 (Ref #: MN985325)

	**WT**	**ORF3-NG**	**NG-ΔORF7a**
gRNA	29870 bp	30578 bp	30221 bp
sgRNA-S	8384 bp	9092 bp	8735 bp
sgRNA-ORF3 (WT/NG)	4555 bp	5263 bp	4906 bp
sgRNA-E	3703 bp	3703 bp	4054 bp
sgRNA-M	3467 bp	3467 bp	3818 bp
sgRNA-ORF6	2744 bp	2744 bp	3095 bp
sgRNA-ORF7 (WT/NG)	2552 bp	2552 bp	2903 bp
sgRNA-ORF8	2052 bp	2052 bp	2052 bp
sgRNA-N	1680 bp	1680 bp	1680 bp

hCoV-OC43 (Ref #: NC_006213.1)

	**WT**	**NG-Δns2**	**NG-ns12.9**
gRNA	30713 bp	30707 bp	31475 bp
sgRNA-ns2 (WT/NG)	9284 bp	9278 bp	10046 bp
sgRNA-HE	8438 bp	8438 bp	9200 bp
sgRNA-S	7140 bp	7140 bp	7902 bp
sgRNA-ns12.9 (WT/NG)	2991 bp	2991 bp	3753 bp
sgRNA-E	2798 bp	2798 bp	2798 bp
sgRNA-M	2409 bp	2409 bp	2409 bp
sgRNA-N	1711 bp	1711 bp	1711 bp

The size includes the leader but excludes the poly-A sequence. The RNA containing the NG sequence is marked in green.

Table S4. Sequence analysis of WT and CPER-generated SARS-CoV-2 and hCoV-OC43 NG viruses recovered from infected cells.

Position	SARS-CoV-2 MN985325	WT	F3-NG	NG-ΔORF7a	Locus	Reference codon	AA reference	Mutated codon	AA change
7486	A	A	U	U	ORF1A	UCA	S	UCU	S
7489	U	U	A	A	ORF1A	ACU	T	ACA	T
18060	U	U	C	C	ORF1AB	CUU	L	CUC	L
26261	C	U/C*	C	C	E	UCG	S	UUG	L
26542	C	U/C*	C	C	M	ACU	T	AUU	I
28853	U	A/U*	U	U	N	UCA	S	ACA	T
**Position**	**hCoV-OC43 AY391777**	**WT**	**NG-Δns2**	**NG-ns12.9**	**Locus**	**Codon**	**AA**	**Mutated codon**	**AA change**
32	C	U	C	C	Leader	N/A	N/A	N/A	N/A
59	U	C/T*	C	C	Leader	N/A	N/A	N/A	N/A
90	C	U	U	U	5 UTR	N/A	N/A	N/A	N/A
530	C	C	C	A/C*	ORF1A	ACC	T	AAC	N
1159	C	U	U	U	ORF1A	CGU	R	UGU	C
1241	C	U/C*	U/C*	U	ORF1A	GCA	A	GUA	V
1925	U	C	C	C	ORF1A	UUU	F	UCU	S
5260	C	U/C*	U/C*	U	ORF1A	CAU	H	UAU	T
6978	G	U	U	U	ORF1A	AUG	M	AUU	I
7673	C	U	U/C*	U	ORF1A	UCU	S	UUU	F
8987	A	G	G	G	ORF1A	AAU	N	AGU	S
11556	A	C	C	C	ORF1A	AGA	R	AGC	S
16841	U	A	A	A	ORF1AB	AGU	S	AGA	R
18385	A	C	C	C	ORF1AB	GAU	D	GCU	A
18570	G	A	A	A	ORF1AB	GGU	G	AGU	S
21992	C	U	N/A	U	ns2	CCC	P	CCU	P
22146	G	U	N/A	U	ns2	GUG	V	UUG	L
23499	U	C	C	C	HE	UUA	L	UCA	S
23501	C	U	U	U	HE	CCC	P	UCC	S
24092	C	U	U	U	S	ACA	T	AUA	I
24103	U	G	G	G	S	UAU	Y	GAU	H
24197	A	G	G	G	S	CAU	H	CGU	R
24370	U	C	C	C	S	UAU	Y	CAU	H
24433	A	G	G	G	S	AAU	N	GAU	D
24650	U	C	C	C	S	CUU	L	CCU	P
24754	A	G	G	G	S	AGC	S	GGC	G
24824	G	C	C	C	S	GGU	G	GCU	A
25928	G	U	U	U	S	AGA	R	AUA	I
26272	C	U	U	U	S	CUU	L	UGU	C
26273	U	G	G	G	S	CUU	L	UGU	C
26585	U	C	C/U*	C	S	CUU	L	CCU	P
26587	U	C	C	C	S	UGG	W	CUG	L
26588	G	U	U	U	S	UGG	W	CUG	L
26602	G	C	C	C	S	GGU	G	CGU	R
26878	C	U/C*	C	U/C*	S	CUU	L	UUU	F
26966	C	A/C*	C	C	S	ACA	T	AAA	K
26997	G	G	C	C	S	AUG	M	AUC	I
27018	C	C	U	U	S	GUC	V	GUU	V
27305	U	G	G	G	S	GUU	V	GGU	G
27513	C	U	U	U	S	AUC	I	AUU	I
27567	A	G	A/G*	G/A*	S	GUA	V	GUG	V
29200	U	G	G	G	N	UUU	F	GUU	V

N Reference   N Mutation   N/N* Polymorphic-major/minor   N/A Not applicable

The A549-ACE2 cells were infected with SARS-CoV-2 WT, ORF3-NG, or NG-ΔORF7a virus with 0.05 MOI. The total cell RNA was extracted at 48 h postinfection using TriPure Reagent. HCT-8 cells were infected with 100 µl of hCoV-OC43 WT, NG-Δns2, and NG-ns12.9 virus inoculum. The viral RNA was isolated from 200 ul of the infected HCT-8 cell culture supernatant harvested 6 dpi using Zymo Quick-RNA Viral Kit. All RNA samples were converted to sequence libraries using TruSeq Stranded Total RNA Kit and sequenced by Illumina MiSeq platform. The obtained RNA reads were mapped to chimeric hg39-SARS-CoV-2 (GenBank Acc. No. MN985325) or hg39-hCoV-OC43 (GenBank Acc. No. AY391777) reference genomes. The consensus sequences of individual viruses were extracted using IGV and aligned with the reference genome using Clustal Omega. The effect of observed mutations on protein-coding potential were determined based on annotated ORF.
